# Male-Biased Autosomal Effect of 16p13.11 Copy Number Variation in Neurodevelopmental Disorders

**DOI:** 10.1371/journal.pone.0061365

**Published:** 2013-04-18

**Authors:** Maria Tropeano, Joo Wook Ahn, Richard J. B. Dobson, Gerome Breen, James Rucker, Abhishek Dixit, Deb K. Pal, Peter McGuffin, Anne Farmer, Peter S. White, Joris Andrieux, Evangelos Vassos, Caroline Mackie Ogilvie, Sarah Curran, David A Collier

**Affiliations:** 1 MRC Social, Genetic and Developmental Psychiatry Centre, Institute of Psychiatry, King’s College London, London, United Kingdom; 2 Department of Cytogenetics, Guy's and St Thomas’ NHS Foundation Trust, London, United Kingdom; 3 Department of Clinical Neuroscience, Institute of Psychiatry, King’s College London, London, United Kingdom; 4 Center for Biomedical Informatics, The Children’s Hospital of Philadelphia, Philadelphia, Pennsylvania, United States of America; 5 Division of Oncology, The Children’s Hospital of Philadelphia, Philadelphia, Pennsylvania, United States of America; 6 Institut de Génétique Médicale, CHRU de Lille, Lille, France; 7 Discovery Neuroscience Research, Eli Lilly and Company Ltd, Lilly Research Laboratories, Erl Wood Manor, Windlesham, Surrey, United Kingdom; University of Illinois at Chicago, United States of America

## Abstract

Copy number variants (CNVs) at chromosome 16p13.11 have been associated with a range of neurodevelopmental disorders including autism, ADHD, intellectual disability and schizophrenia. Significant sex differences in prevalence, course and severity have been described for a number of these conditions but the biological and environmental factors underlying such sex-specific features remain unclear. We tested the burden and the possible sex-biased effect of CNVs at 16p13.11 in a sample of 10,397 individuals with a range of neurodevelopmental conditions, clinically referred for array comparative genomic hybridisation (aCGH); cases were compared with 11,277 controls. In order to identify candidate phenotype-associated genes, we performed an interval-based analysis and investigated the presence of ohnologs at 16p13.11; finally, we searched the DECIPHER database for previously identified 16p13.11 copy number variants. In the clinical referral series, we identified 46 cases with CNVs of variable size at 16p13.11, including 28 duplications and 18 deletions. Patients were referred for various phenotypes, including developmental delay, autism, speech delay, learning difficulties, behavioural problems, epilepsy, microcephaly and physical dysmorphisms. CNVs at 16p13.11 were also present in 17 controls. Association analysis revealed an excess of CNVs in cases compared with controls (OR = 2.59; p = 0.0005), and a sex-biased effect, with a significant enrichment of CNVs only in the male subgroup of cases (OR = 5.62; p = 0.0002), but not in females (OR = 1.19, p = 0.673). The same pattern of results was also observed in the DECIPHER sample. Interval-based analysis showed a significant enrichment of case CNVs containing interval II (OR = 2.59; p = 0.0005), located in the 0.83 Mb genomic region between 15.49–16.32 Mb, and encompassing the four ohnologs NDE1, MYH11, ABCC1 and ABCC6. Our data confirm that duplications and deletions at 16p13.11 represent incompletely penetrant pathogenic mutations that predispose to a range of neurodevelopmental disorders, and suggest a sex-limited effect on the penetrance of the pathological phenotypes at the 16p13.11 locus.

## Introduction

Copy number variants (CNVs) represent one the most prevalent types of structural variations detected in the human genome and are a major source of human genetic variability [1]–[4]. Many rare CNVs are associated with pathological conditions, including classic “genomic disorders”, such Williams-Beuren syndrome (deletion at 7q11.23; OMIM 194050), as well as those with a complex genetic aetiology such as schizophrenia and autism spectrum disorders [5]–[8]. Many of these complex disorders show a distorted sex ratio, usually with an excess of affected males. In autism spectrum disorders the male: female ratio is usually quoted at about 4∶1, but may be higher [9], and a significant male excess is also found in schizophrenia, albeit less pronounced at about 1.4∶1 [10]. The reasons for this are unknown, but appear to be largely autosomal in origin and could result from a variety of factors, including imprinting, endocrine or neurodevelopmental factors, which result in a categorical protection from expression in females (resilience) [11]. A better understanding might provide insight for the development of treatments. The identification and characterisation of specific autosomal loci showing sex-biased effects will be an important step in this direction.

Putatively pathogenic CNVs may arise *de novo* or be inherited, and many show a pleiotropic effect, with broad phenotypic manifestations and incomplete penetrance both overall and for specific disorders [12]. An important genomic “hotspot” for these variants is the 16p13.11 locus, which is especially rich in low-copy repeats (LCRs). These highly homologous DNA sequences are characteristically involved in non-allelic homologous recombination (NAHR), a major source of *de novo* genomic rearrangements in human [13], [14]. Deletions and reciprocal duplications at the 16p13.11 locus have been associated with disorders involving neurodevelopment, including conditions such as autism, mental retardation, schizophrenia and epilepsies, which appear to share overlapping genetic aetiologies [15]–[25]. Pathogenic genomic variants at 16p13.11 were initially described by Ullmann et al. [15], who identified a 1.5 Mb duplication (14.89–16.39 Mb) and a reciprocal deletion spanning the same interval, in four severe autistic patients from three unrelated families, and in three unrelated intellectually disabled patients. In the affected subjects, some of the imbalances were inherited from unaffected or mildly affected parents, indicating incomplete penetrance [15]. In a subsequent study, Hannes et al. identified six deletions and seven duplications at the 16p13.11 locus in thirteen unrelated patients affected by intellectual disability and/or multiple congenital anomalies; five deletions and five duplications spanned approximately the same 1.5 Mb region identified in the first report, whilst the other three patients carried atypical larger rearrangements, including two duplications of ∼3.4 Mb in size (15.1–18.5 Mb), overlapping the typical 1.5 Mb region, and a deletion of ∼1.6–2.1 Mb (16.3–18.4 Mb), flanking the region. Of the variations identified, one duplication occurred *de novo*, two deletions and two duplications were inherited from an apparently unaffected parent, and one duplication was inherited from a mildly affected mother [16].

CNVs at 16p13.11 identical or similar to the variants originally described have subsequently been identified in several cohorts of patients affected by schizophrenia, epilepsies, attention-deficit hyperactivity disorder (ADHD), developmental delay (DD), dysmorphic features and multiple congenital anomalies and, most recently, thoracic aortic aneurysms and dissections (TAAD) [15]–[25]. Significant sex differences in prevalence, course and severity have been described for a number of these conditions but the biological and environmental factors underlying these sex-specific features remain unclear. Among the possible biological factors, an important role is thought to be played by sex-hormone levels, which differ between males and females, and are particularly relevant during fetal and neonatal development, acting on the brain to produce sex differences in behaviour, cognition, brain structure and function [26].

Genetic factors are likely to represent another major determinant of phenotypic dimorphism [27]. A sex-specific genetic architecture has been observed for a variety of quantitative and disease-related traits and recent studies suggest that, together with the well established contribution of the genes on the sex chromosomes, natural variation within the autosomal genome also plays an important role in the aetiology of these sexually dimorphic features; in this context, sex could be considered as an environmental factor that interacts with the autosomal genes and modulates penetrance and expressivity of a number of traits [28]. Furthermore, the sex-specific autosomal effects are likely to be in part explained by factors that are not directly associated with nucleotide sequence changes and that operate at the interface between the genome and the environment, such as differences in gene-expression or epigenetic processes; these factors underlie important phenotypic differences between the sexes, and have therefore the potential, if perturbed, to convert such differences in sex-specific susceptibility to disease [27], [29].

In the present study, we investigated the burden and the possible sex-biased effect of CNVs in the 16p13.11 region in a UK sample of 10,397 children and young adults with a childhood onset neurodevelopmental condition, referred for clinical genetic testing by array comparative genomic hybridisation (aCGH), using a 60 K Agilent oligonucleotide platform.

## Materials and Methods

### Ethics Statement

The BBGRE project was approved by the Cambridgeshire Central Research Ethics Committee. Under common UK law all research using identifiable personal data requires the express consent of the individuals involved. The use of non-identifiable information does not fall under common law, i.e. informed consent is not necessary for research using this type of data, when it is irreversibly anonymised. This is specified in the UK Data Protection Act (DPA) (1998), which came into force in 2000, in an exemption clause for research using unlinked anonymised data: “Informed consent is not necessary for research which makes use of unlinked anonymised data”. Since the present study uses only unlinked anonymised data, their use was therefore in accordance with the UK Data Protection Act (DPA) (1998). See http://www.legislation.gov.uk/ukpga/1998/29/contents for further information.

### Clinical Diagnostic Referral Cases

The clinical cytogenetic sample consisted of patients referred to Guy’s and St Thomas NHS Foundation Trust from regional paediatricians and other health specialists, as well as from genetics centres both in and outside the region (South-East Thames). Array CGH analysis was performed to determine if there were detectable structural genetic abnormalities that could be of aetiological significance for a range of problems including developmental delay (DD), intellectual disability (ID), autism spectrum disorders (ASD), attention-deficit hyperactivity disorder (ADHD), specific developmental delays such as speech or language delay, birth defects or to confirm a clinical diagnosis of a suspected syndrome. All patient tests were carried out as part of standard clinical care, either as clinical referrals for array CGH testing following a normal karyotype, or those having array CGH as a first-line test in place of karyotyping. All data were anonymised.

### Array CGH Analysis

Testing was carried out at a cytogenetics CPA accredited laboratory. DNA samples were analyzed by array comparative genomic hybridisation (aCGH), using a 60 K Agilent array (designs 028469 and 017457), with a total imbalance detection rate of 24%. Array CGH analysis was performed as described previously [30]. In brief, DNA extracted from blood samples (1 µg) was labelled using CGH Labelling Kit for Oligo Arrays (Enzo Life Sciences, USA), then applied to a 60 K oligonucleotide array (Agilent, USA). Image quantification, array quality control and aberration detection were performed using Feature Extraction and DNA Analytics software packages (Agilent, USA) for oligo arrays, according to the manufacturer’s instructions. All copy number variations in the 16p13.11 region (46/46) were confirmed either by a second CGH array with a different comparison sample or by a multiplex ligation-dependent probe amplification assay (MLPA kits P064, P245 and custom probes; MRC-Holland, Netherlands) [30], [31]. Genomic data and referral phenotype information was recorded in a clinical database, which at the time of analysis contained 10,397 clinical referrals (63.4% males; October 2012), including approximately 1,400 patients referred for ASD, 26% of whom were female. The overall ethnic distribution (self-reported data) was 70% Caucasians, 15% Africans and 15% other/mixed ancestry. Copy number variants in this population are available in the Brain and Body Genetic Resource Exchange database (BB-GRE; http://bbgre.org).

### Controls

We analysed a total sample of 6,078 control individuals, including 459 controls (281 female, 178 male) comprehensively screened for a lifetime absence of psychiatric disorder and 5,619 controls (2,611 female, 2,668 male) from the Wellcome Trust Case-Control Consortium Phase II (WTCCC2). Screened controls were recruited from students and staff at King’s College London; subjects were interviewed with a modified version of the Past History Schedule [32] and with the Beck Depression Inventory [33] and were included only if they demonstrated no evidence of past or present psychiatric disorder. The WTCCC2 control cohort consists of the UK 1958 British birth cohort (58 BC) and a cohort derived from the UK national blood service (NBS) [34]. Both cohorts represent population control samples. DNA samples were derived from immortalised cell lines (58 BC cohort) and venous blood (screened controls and NBS cohort). Screened control samples were genotyped on the Illumina HumanHap 610-Quad beadchip, whereas WTCCC2 samples were genotyped on a modified Illumina 1 M beadchip. To ensure comparable CNV detection from different array types, control CNVs were identified using a consensus marker set between the 610 K and 1 M chips, which mimics the Illumina HumanHap 550 beadchip (detailed description of data analysis and quality control criteria can be found in the Methods S1 and in Rucker et al. [34]).

Additionally, we used control data from two previously published studies by Shaikh et al. and Cooper et al. [35], [36]. The Shaikh control sample consists of 2,026 healthy children (1,104 female, 922 male) recruited within the Children’s Hospital of Philadelphia (CHOP) health care network, uniformly genotyped using the Illumina HumanHap 550 beadchip, with DNA samples obtained from whole blood, (overall precision >96% in identifying CNVs represented by >10 probes) (http://cnv.chop.edu) [35]. The Cooper control sample originally consisted of 8,329 individuals, but we used genomic information only for the subset of 3,173 subjects (2,148 female, 1,025 male) for which gender data were available. DNA was obtained from cell lines (HGDP, n = 984), and blood samples (London, n = 760; FHCRC, n = 1,429), and genotyping was performed using the Illumina HumanHap 550 K, 650 Y and 610-Quad beadchips, with an overall precision >89% in identifying CNVs >100 kbp (dbVar, nstd54) [36].

Ethnic distribution in the total control population (n = 11,277) was 77% Caucasians, 8.5% Africans and 14.5% other/mixed ancestry. Although the control sample was slightly enriched in Caucasians (77% vs 70%) and had slightly fewer African individuals (8.5% vs 15%) compared to the case sample, we found no significant differences in the frequency of 16p13.11 CNVs in the Caucasian and African control subgroups (OR = 1.78; p = 1.0), which suggests no potential population stratification problems.

Despite 33% of DNA samples in our control population were derived from immortalized cell lines (58 BC and HGDP cohorts), the CNV QC measures used in both the WTCCC2 and Cooper et al. control cohorts were designed to account for potential cell line mosaicism and artefacts (Methods S1) [34], [36], [37]. Moreover, in order to further test for DNA source effects, we also split the total control sample and compared the frequency of samples with 16p13.11 CNVs in the whole blood (0.14%) and cell line (0.20%) subgroups, but we found no significant differences (OR = 1.39; p = 0.498), which suggests that cell-line artefacts are not a major contributor to our estimates of CNV burden.

### Association Analysis

Pearson’s chi-square test or Fisher’s exact test, as appropriate, were used to compare frequencies of NAHR-mediated CNVs in cases and controls and to compare interval copy number frequencies in case and control CNVs. All data analysis was performed using the R language and environment for statistical computing (http://www.r-project.org/).

Despite the platform heterogeneity in CNV detection, numerous studies have shown that, whereas for small variations the CNV detection power is dependent on the array used [38], different array platforms have comparable sensitivity and specificity for the detection of large CNVs (>500 kbp in size), and ten probes are typically sufficient to detect such events [37], [39], [40]. Since the 16p13.11 duplications and deletions are large NAHR-mediated variations with size >800 kbp, and given that the different array platforms used for their detection in cases and controls have an adequate probe coverage for the 16p13.11 region (Methods S1), platform-specific differences in detection are unlikely to represent a major confounding factor in our analyses.

### DECIPHER Database Search

We performed a search in the DECIPHER database (https://decipher.sanger.ac.uk), in order to identify additional cases with CNVs in the 16p13.11 region. DECIPHER (Database of Chromosomal Imbalance and Phenotype in Humans Using Ensembl Resources) is an interactive web-based database that collects clinical phenotype information and copy number change data from a sample of >18,000 patients suffering from developmental disorders, enabling clinical scientists worldwide to maintain records for their patients and to share this information with the clinical research community [41]. At the time of analysis (October 2012), the DECIPHER database contained 18,451 clinical referrals, 54% of whom were male.

### Evolutionary Genetic Analysis

Ohnologs in the 16p13.11 region were defined as described by Makino and McLysaght [42]. Ohnologs were syntenic genes located on paralogous chromosomal regions and derived from whole genome duplication (WGD). A list of ohnologs in the region was provided for the maximal CNV interval described in the present study (Chr16∶14.66–18.70 Mb, GRCh37/hg19), blind to the coordinates of individual CNVs and to interval-based analyses (Makino and McLysaght, personal communication).

## Results

In the case series of 10,397 individuals, we identified 46 patients with copy number variants of variable size at the 16p13.11 locus, including 28 duplications (all NAHR-mediated) and 18 deletions (16 NAHR-mediated and 2 non-NAHR mediated), all contained within the region between 14.66 and 18.70 Mb (Human Genome Build 37), where the previously reported copy number variants have been described [15]–[25].

**Figure 1:**
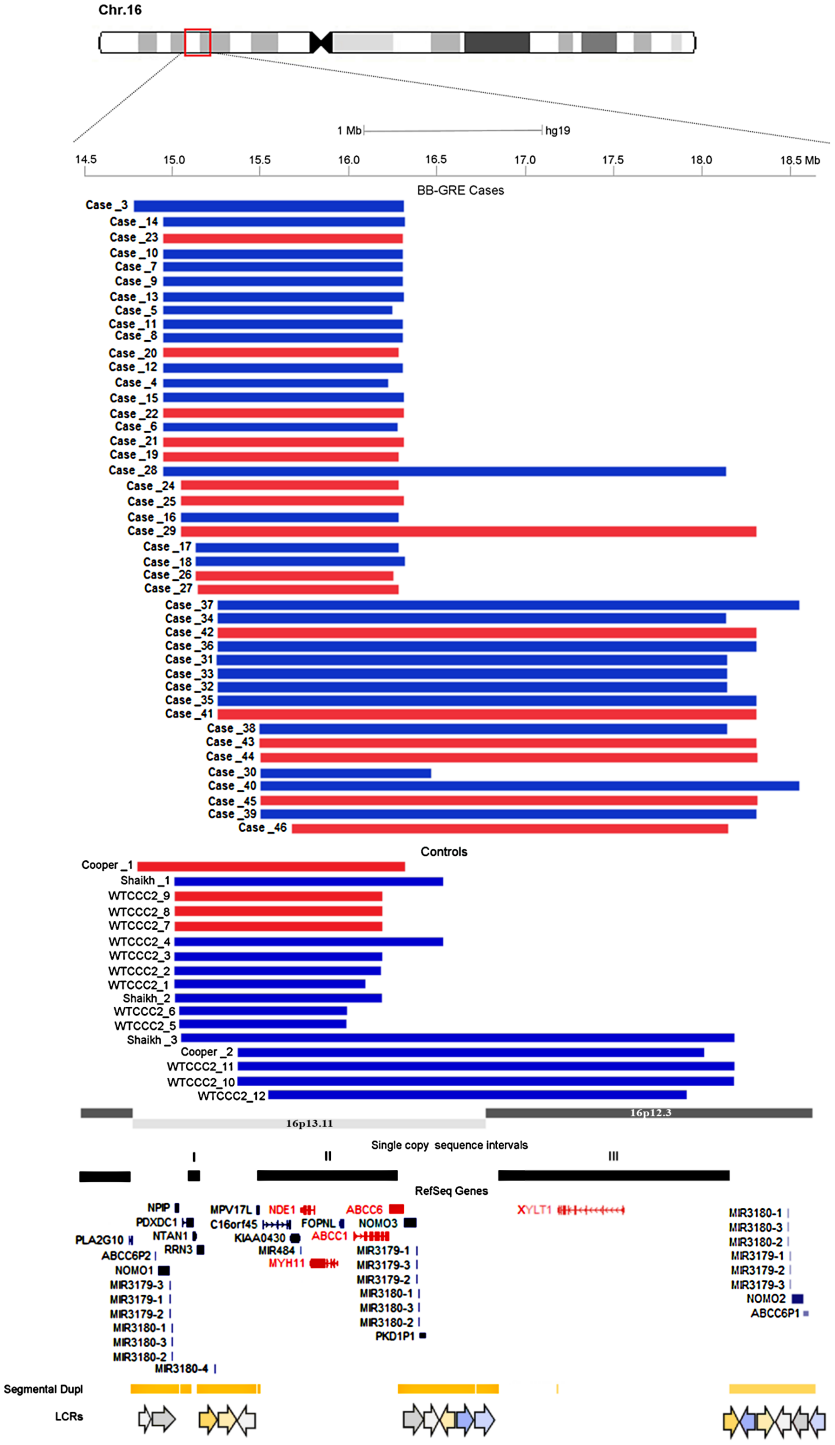
NAHR-mediated duplications and deletions of 16p13.11. NAHR-mediated duplications (blue) and deletions (red) identified in the 16p13.11–p12.3 region (Chr16∶14.66–18.70 Mb, GRCh37/hg19) in cases and controls; case and control IDs refer to Table S1 and Table S2. Black solid bars indicate the three single copy sequence intervals in the region. Red and blue gene symbols represent ohnologs and other genes respectively. Segmental duplications and low copy repeats (LCRs) in the region are also shown.

Given the high number of variable sized CNVs, for their classification we referred to the study by Ingason et al. [19], who subdivided the 16p13.11 region into three single copy sequence intervals called interval I, II and III, each flanked by sequences rich in low copy repeats (LCRs). We identified seven distinct categories of NAHR-mediated CNVs in the region, including nine deletions and sixteen reciprocal duplications of intervals I and II, one deletion and one duplication of intervals I, II and III, one duplication of interval II, six deletions and ten duplications of intervals II and III (Figure 1, Table 1, Table S1); furthermore, we identified two cases carrying identical, non-NAHR mediated 23 kbp microdeletions encompassing the NTAN1 gene, and these were excluded from the analyses (Figure S1, Table S1).

**Table 1:** NAHR-mediated duplications and deletions of 16p13.11 identified in the BB-GRE referral cases.

	Total Sample		Male only		Female only	
CNV	Cases (N = 10,397)*	Controls (N = 10,375)	Cases (N = 6,595)	Controls (N = 4,474)	Cases (N = 3,802)	Controls (N = 5,901)
All Dupl	28	13	20	4	7	9
All Del	16	4	13	0	3	4
Dupl I	0	0	0	0	0	0
Dupl I+II	16	8	11	3	5	5
Dupl I+II+III	1	1	0	0	0	1
Dupl II	1	0	1	0	0	0
Dupl II+III	10	4	8	1	2	3
Del I	0	0	0	0	0	0
Del I+II	9	4	7	0	2	4
Del I+II+III	1	0	1	0	0	0
Del II	0	0	0	0	0	0
Del II+III	6	0	5	0	1	0

Abbreviations: Dupl, duplication; Del, deletion.

* The sex of one of the cases carrying a duplication of intervals I, II and III was unknown, consequently, we were unable to include him/her in the male or female subgroup.

Within our two control groups, 348 screened controls and 4,828 unscreened controls passed quality control criteria and were thus included in the analysis. We did not detect any 16p13.11 copy number variation in the screened controls, whereas we identified twelve NAHR-mediated CNVs in the WTCCC2 control groups, including three deletions and nine duplications; additionally, three NAHR-mediated duplications were identified in the Shaikh controls, while one deletion and one duplication were identified in the Cooper control group, with a total control CNV count of 17 CNVs and four distinct CNV categories (Figure 1, Table 1, Table S2).

Association analysis showed a significant enrichment of NAHR-mediated CNVs in cases compared with controls (OR = 2.59; p = 0.0005), with the effect coming mostly from deletions (OR = 4.00; p = 0.007), that were present in 0.15% of cases versus 0.04% of controls; duplications were also more common in the cases, being identified in 0.27% of cases versus 0.12% of controls (OR = 2.15; p = 0.019) (Table 2). Interestingly, performing a post hoc sex-specific analysis, we observed a significant enrichment of CNVs (OR = 5.62; p = 0.0002), coming from duplications (OR = 3.40; p = 0.018) and, particularly, from deletions (OR = ∞; p = 0.001), only in the male subgroup of cases, but not in females (All CNVs: OR = 1.19, p = 0.673; Del: OR = 1.16, p = 1.00; Dupl: OR = 1.21, p = 0.708), in line with our preliminary hypothesis of a possible sex-biased effect for the 16p13.11 CNVs. Finally, since some of the cases with a 16p13.11 CNV also harboured a second potentially pathogenic chromosomal imbalance, we performed a post hoc analysis, excluding cases (7/44) and controls (3/17) carrying additional very rare second-hit CNVs >500 kpb in size (comparable sensitivity and specificity between aCGH and SNP arrays) (Table S1, Table S2). This additional analysis confirmed all the results described above, both overall (OR = 2.64; p = 0.0013), and in the male (OR = 6.35; p = 0.00048) and female subgroups (OR = 1.13; p = 0.794).

**Table 2:** Frequency of NAHR-mediated 16p13.11 duplications and deletions in BB-GRE cases and controls.

16p13.11 CNV	Cases (%)	Controls (%)	OR (95% CI)	p-value
All	44 (0.42)	17 (0.16)	2.59 (1.48–4.53)	0.0005
Duplications	28 (0.27)	13 (0.12)	2.15 (1.11–4.16)	0.019
Deletions	16 (0.15)	4 (0.04)	4.00 (1.34–11.96)	0.007

Abbreviations: CNV, copy number variant; OR, odds ratio; CI, confidence interval.

In an attempt to further confirm our findings, we next tested for a sex-biased prevalence of the variations also in the DECIPHER sample. The DECIPHER database search identified 101 patients with NAHR-mediated CNVs at the 16p13.11 locus, including 61 duplications and 40 deletions, with nine distinct CNV categories (Figure S2, Table S3, Table S4). Overall, we observed a significant enrichment of CNVs in the cases compared with controls (OR = 3.35; p = 9.8×10^−7^), with the effect coming from both deletions (OR = 5.63; p = 0.0002) and duplications (OR = 2.64; p = 0.0009). Sex-specific analysis confirmed a significant overrepresentation of deletions (OR = ∞; p = 0.0003) and duplications (OR = 3.60; p = 0.0098) only in the male subgroup of cases but not in females (Dupl: OR = 2.09, p = 0.051; Del: OR = 1.91, p = 0.26). All the results were confirmed when we excluded from the analysis cases (18/101) and controls (3/17) harbouring additional rare second-hit CNVs >500 kpb in size (Overall: OR = 3.34, p = 9.4×10^−6^; Males: OR = 7.97, p = 3.2×10^−5^; Females: OR = 1.84, p = 0.082).

Given the very variable size of the genomic variations identified, we next decided to perform a post hoc interval-based analysis, comparing the frequencies of the three single copy sequence intervals contained in our region of interest in case and control CNVs, in order to identify a possible core pathogenic region, harbouring the dosage-sensitive genes responsible for the deleterious phenotypes observed in our cases.

Interval-based analysis revealed a significant enrichment of case CNVs containing interval II (OR = 2.59; p = 0.0005), which is located in the 0.83 Mb genomic region between 15.49 and 16.32 Mb (Human Genome Build 37) and encompasses a core set of 9 genes (MPV17L, C16orf45, KIAA0430, NDE1, MIR484, MYH11, C16orf63, ABCC1, ABCC6); the interval II overrepresentation was observed for both case deletions (OR = 4.00; p = 0.007) and duplications (OR = 2.15; p = 0.019). In line with the sex-specific analysis results described above, performing the analysis in the male and female subgroups, we found a significant enrichment of CNVs containing interval II (OR = 5.62; p = 0.0002), coming from both deletions (OR = ∞; p = 0.001) and duplications (OR = 3.40; p = 0.018), only in the male subgroup of cases, but not in females (OR = 1.19; p = 0.673). The same pattern of results was also observed in the DECIPHER sample, both overall (Dupl: OR = 2.60, p = 0.001; Del: OR = 5.49, p = 0.0003) and in the two subgroups (Male: Dupl: OR = 3.60, p = 0.0098; Del: OR = ∞, p = 0.0004. Female: Dupl: OR = 2.01, p = 0.066; Del: OR = 1.91, p = 0.26).

Finally, we applied an evolutionary genetic approach, and tried to identify the disease-causing genes in the 16p13.11 region through the mapping of ohnologs, genes retained after ancestral whole genome duplication events, that are inferred to be particularly enriched in dosage-sensitive genes [42]. We identified a total of five ohnologs in the region, including NDE1, MYH11, ABCC1, ABCC6 and XYLT1 (Figure 1, Table 3); interestingly, four of the ohnologs (NDE1, MYH11, ABCC1, ABCC6) were part of the core set of genes located in interval II, most overrepresented in our case CNVs and are thus likely to represent dosage-balanced ohnologs, involved in the aetiology of the pathological phenotypes associated with the 16p13.11 copy number variants.

**Table 3:** Ohnologs in the 16p13.11–p12.3 region.

Ensembl id	Gene symbol	Full name
ENSG00000072864	NDE1	NudE nuclear distribution gene E homolog 1
ENSG00000133392	MYH11	Myosin heavy chain 11
ENSG00000103222	ABCC1	ATP-binding cassette, sub-family C, member 1
ENSG00000091262	ABCC6	ATP-binding cassette, sub-family C, member 6
ENSG00000103489	XYLT1	Xylosyltransferase 1

### 16p13.11 CNVs in Neurodevelopmental Disorders

Phenotypic information was available for 41 of the 46 patients with a copy number variation at 16p13.11, identified in the clinical referral series (Table S1). Of them, the only patient not diagnosed with a neurodevelopmental condition was a ten months old male carrying a deletion of intervals II and III, who was referred for testing as part of developmental screen and documented as having a bullous skin condition (‘scalded-skin syndrome’). However, given the very young age at referral (<1 year), and since the deletion was inherited from a father with learning difficulties, it is possible that a neurodevelopmental disorder will later manifest also in this patient.

We were able to investigate the inheritance pattern of the variations within the family trio (father, mother and affected child) for twenty-six of the carrier patients. One duplication (male proband) and four deletions (three males and one female) arose *de novo*, whereas twenty-one variations were inherited from a parent, including fourteen duplications (six maternal and eight paternal) and seven deletions (five maternal and two paternal).

We identified two patients carrying identical non-NAHR mediated microdeletions of NTAN1; both the variations arose *de novo* and have been identified in a ten years old male referred for autism spectrum disorder (ASD) and learning difficulties, and in a five years old male referred for attention difficulties, delayed speech and language skills and dysmorphic features. The deletions involve a 23 kbp genomic interval (chr16∶15,131,723–15,154,746) and encompass the entire NTAN1 gene, one of the strongest candidate genes in the 16p13.11 region (Figure S1). NTAN1 encodes the asparagine-specific N-terminal amidase, an enzyme involved in the regulation of the *in vivo* half life of proteins; inactivation of the NTAN1 gene in mice has been associated with abnormal neurological features such as altered social behaviour and impaired spatial and non-spatial learning and memory [43]–[45]. Interestingly, whilst the patient referred for attention difficulties, speech delay and dysmorphic features also harboured a maternally inherited, putatively pathogenic duplication, affecting part of the IL1RAPL1 gene (Xp21.2), the patient with a specific diagnosis of ASD and learning difficulties did not carry any additional genomic imbalance, which could suggest the involvement of the NTAN1 gene in the aetiology of the neurodevelopmental disorder.

Patients with NAHR-mediated duplications, the larger group identified, presented with a wide range of abnormal phenotypes. Consistent with the ascertainment criteria, we observed developmental delay (DD) (12/24) and dysmorphic features (n = 11) in most cases; importantly, the majority of patients older than 2 years (14/18) presented with a specific neurologic/neuropsychiatric phenotype, including autism spectrum disorder (ASD) (n = 4), speech and language delay (n = 5), learning difficulties (n = 3), behavioural problems (n = 2), microcephaly (n = 2), epileptic seizure (n = 1) and attention deficit hyperactivity disorder (ADHD) (n = 1). Other relevant clinical features included cardiac anomalies (n = 2), motor delay (n = 1) and obesity (n = 1) (Table 4, Table S1). We also identified a mildly affected carrier mother with speech delay. There were no notable phenotypic differences among carriers of duplications with different size. Additional genomic imbalances were identified in six of the 28 probands, including a male patient referred for ASD, harbouring an additional maternally inherited duplication at 6q21, a region with reported linkage to schizophrenia and bipolar disorder [46], [47], a female patient diagnosed with DD and microcephaly carrying an additional duplication at 22q11.22q11.23, recently associated with DD, hyperactivity, and epilepsy [48], and a proband harbouring an additional paternally inherited deletion of uncertain significance at 6p22.2. The other three duplication carriers with an additional genomic imbalance included a male patient referred for DD and physical dysmorphisms, harbouring an additional duplication at 7q11.23, the Williams-Beuren Syndrome critical region, recently associated with autism [49], a female patient referred for DD, microcephaly and physical dysmorphisms presenting with a complex chromosome 7 rearrangement, and a male patient diagnosed with ASD or Asperger syndrome carrying a duplication of uncertain significance at Xp22.33, flanking the SHOX gene.

**Table 4:** Frequency of phenotypic features in individuals with 16p13.11 duplications and deletions.

Phenotypes	Duplications				Deletions			
	BB-GRE	Decipher	Overall	%	BB-GRE	Decipher	Overall	%
Developmental delay	12/24	43/49	55/73	75.3%	8/14	25/29	33/43	76.7%
Dysmorphic features	11/24	24/49	35/73	47.9%	5/14	12/29	17/43	39.5%
Speech delay*	5/18	20/37	25/55	45.4%	3/8	8/20	11/28	39.3%
Learning difficulties*	3/18	19/35	22/53	41.5%	6/8	5/19	11/27	40.7%
Psychiatric/Behavioural problems **°** *	7/18	17/35	24/53	45.3%	5/8	4/19	9/27	33.3%
Seizures*	1/18	3/35	4/53	7.5%	2/8	7/20	9/28	32.1%
Microcephaly*	2/18	4/35	6/53	11.3%	3/8	5/20	8/28	28.6%
Congenital anomalies	4/24	14/49	18/73	24.7%	2/14	12/29	14/43	32.6%
Motor delay*	1/18	14/35	15/53	28.3%	3/8	9/21	12/29	41.4%
Obesity*	1/18	6/35	7/53	13.2%	-	1/20	1/28	3.6%

**°** This phenotype **i**ncludes the diagnosis of behavioural problems, ADHD and autism spectrum disorder.

ADHD was present only in duplication carriers (11.3%), but not in deletion carriers.

* Not evaluated in very young cases.

The majority of patients with NAHR-mediated deletions presented with developmental delay (8/14); importantly, all patients older than 2 years (8/8) manifested a specific neurologic/neuropsychiatric phenotype, including learning difficulties (n = 6), speech and language delay (n = 3), behavioural problems (n = 3), microcephaly (n = 3), autism spectrum disorder (n = 2) and epilepsy (n = 2). Other relevant abnormal phenotypes included dysmorphic features (n = 5), motor delay (n = 3) and cardiac anomalies (n = 1) (Table 4, Table S1). We also identified a carrier father mildly affected with learning difficulties. There were no notable phenotypic differences among carriers of deletions with different size. Additional genomic imbalances were identified in five of the 16 probands, including a male patient referred for motor delay, harbouring an additional maternally inherited deletion at 17q23.3, encompassing the Silver-Russell syndrome candidate gene CSH1, a male patient diagnosed with DD and microcephaly carrying an additional deletion at 7q11.22, involving the entire autism susceptibility candidate 2 (AUTS2) gene, a female patient referred for ASD, motor delay, microcephaly and physical dysmorphisms harbouring a maternally inherited deletion of uncertain significance at 17q21.32, encompassing part of the C17orf57 gene, and a male patient diagnosed with DD, motor delay and speech delay carrying a duplication of uncertain significance at 4q13.3. The other deletion carrier presenting with an additional genomic imbalance was the male proband referred for scalded-skin syndrome, who harboured an additional maternally inherited deletion at 13q12.12, encompassing the TNFRSF19 gene, in a region with reported linkage to epilepsy [50] (Table S1).

### 16p13.11 CNVs in DECIPHER

Performing a search in the DECIPHER database (https://decipher.sanger.ac.uk) [41], we identified 101 subjects with NAHR-mediated CNVs at the 16p13.11 locus, including 61 duplications and 40 deletions (Figure S2, Table S4). Phenotypic information was available for 78 of the 101 patients (49 duplication carriers and 29 deletion carriers), and between them, seven cases (patients 250947, 250061, 1230, 249225, 993, 4598, 250436) were not diagnosed with a neurodevelopmental disorder, but were referred for physical dysmorphism and congenital anomalies. Information about the inheritance pattern of the variations was available for seventy-one family trios. Five duplications (3 males, 1 female, and 1 unknown sex) and seven deletions (6 males and 1 female) arose *de novo*, whereas fifty-nine variations were inherited from a parent, including forty duplications (24 maternal, 11 paternal, and 5 unknown sex) and nineteen deletions (8 paternal, 6 maternal, and 5 unknown sex). Twenty-seven of the 101 patients (19 duplication carriers and 8 deletion carriers) harboured one or more additional genomic imbalances, either clinically significant (37%) or of uncertain clinical significance (63%) (Table S4).

Notably, the DECIPHER database search also identified two patients (250140 and 265264) carrying non-NAHR mediated 23 kbp microdeletions encompassing the NTAN1 gene (chr16∶15,131,723–15,154,746) identical to those we found in two cases of the referral series (Figure S1). Phenotypes of the patients and inheritance pattern of the variations were unknown, and both probands harboured a second genomic imbalance of uncertain significance (dupl 2q13 and del 12q13.3) (Table S4).

Patients with duplications mostly presented with developmental delay (43/49) and physical dysmorphism (n = 24). In line with our clinical referral cases, the majority of patients older than 2 years (29/35) were also diagnosed with a neurologic/neuropsychiatric phenotype, including speech and language delay (n = 20), learning difficulties (n = 19), autism spectrum disorder (n = 6), behavioural problems (n = 6), ADHD (n = 5), microcephaly (n = 4) and seizures (n = 3). Other important phenotypic features observed in more than one duplication carrier included motor delay (n = 14), congenital anomalies (n = 14) and obesity (n = 6) (Table 4). Seventeen carrier parents (9 mothers, 7 fathers, and 1 unknown sex) were also mildly affected with developmental delay and speech delay, while one carrier mother was severely affected with speech delay and learning difficulties. Twenty-two parents (14 mothers, 4 fathers, and 4 unknown sex) harbouring the duplications were apparently unaffected (Table S4).

The majority of patients with deletions presented with developmental delay (25/29); neurologic/neuropsychiatric phenotypes observed in patients older than 2 years (15/20) included speech and language delay (n = 8), seizures (n = 7), learning difficulties (n = 5), microcephaly (n = 5), autism (n = 3), macrocephaly (n = 2) and behavioural problems (n = 1). Other relevant abnormal phenotypes included physical dysmorphism (n = 12), congenital anomalies (n = 12) and motor delay (n = 9) (Table 4). Seven carrier parents (3 mothers and 4 fathers) were also mildly affected with developmental delay and speech delay, while twelve parents (3 mothers, 4 fathers, and 5 unknown sex) with the same deletions were apparently unaffected (Table S4).

## Discussion

In the present study, we report evidence for a male-biased autosomal effect of 16p13.11 duplications and deletions in a sample of 10,397 individuals with a neurodevelopmental condition, analysed by whole-genome array comparative genomic hybridisation (aCGH). The CNVs identified included 28 duplications with size ranging from 0.8 Mb to 3.29 Mb and 18 deletions with size between 0.02 Mb and 3.26 Mb (2 non-NAHR mediated), each encompassing one or more of the three genomic intervals (I, II and III) previously identified at this locus [19]. The male-biased prevalence of the 16p13.11 variations was also confirmed in the DECIPHER sample, where we identified 101 patients with NAHR-mediated CNVs at the 16p13.11 locus, including 61 duplications and 40 deletions.

Both duplications and deletions were associated with a wide range of phenotypic manifestations. Consistent with the ascertainment criteria, the most common phenotypes observed were developmental delay/learning disability and physical dysmorphism, however, the majority of patients older than 2 years also presented with a specific neurologic/neuropsychiatric phenotype, including autism spectrum disorders, speech and language delay, seizures, behavioural problems, microcephaly and attention deficit hyperactivity disorder. Notably, in line with previous reports, seizures were most commonly observed among deletion carriers (32.1% vs 7.5%) [20]–[22], while ADHD was only present in duplication carriers (11.3%) [23].

Information about the inheritance pattern of the variations was available for twenty-six families of our clinical referral series and seventy-one DECIPHER families; overall, 17.5% of the probands harboured a de novo event, while 82.5% inherited the variation from a carrier parent. Interestingly, we observed an enrichment of de novo duplications and deletions in affected males (13 de novo events in affected males vs 3 de novo events in affected females, p value = 0.0004), consistent with purifying selection and thus higher penetrance of the variations in the male gender. Phenotypic information was available for sixty-one carrier parents; of them, one mother harbouring a duplication, was severely affected with developmental delay, speech delay and learning difficulties, while the remaining carrier parents were only mildly affected (n = 26), or completely unaffected (n = 34). The present data thus suggest that duplications and deletions at chromosome 16p13.11 represent pathogenic variants with incomplete penetrance, a pattern frequently observed for CNVs associated with complex genetic disorders [51]. Notably, we also observed a trend toward a parent-of-origin effect, with carrier mothers being more likely than fathers to transmit a CNV to an affected proband (31 carrier mothers vs 20 carrier fathers, p value  = 0.029).

Twelve patients of the referral series (26.1%) and twenty-seven DECIPHER patients (26.7%) also harboured a second genomic imbalance that was either clinically significant or of uncertain clinical significance. These additional CNVs might act in concert in a “two-hit” model with the 16p13.11 variants to increase penetrance or severity; notably, the “double-hit” model was first proposed for patients with deletions at 16p12.1, a region located in close proximity to the 16p13.11 locus, and the frequency of cases with a second-hit CNV in the present study is very similar to that observed in the original report [40].

Association analysis showed a significant enrichment of NAHR-mediated CNVs in the cases compared with controls, with the strongest overrepresentation coming from deletions. In line with our preliminary hypothesis, we observed a sex-biased effect, with a significant enrichment of CNVs only in the male subgroup of cases, also confirmed in the DECIPHER sample. All the findings also held after excluding from the analysis cases and controls with rare second-hit CNVs >500 kpb in size. Assuming an equal incidence of the 16p13.11 imbalances in males and females, our data clearly suggest a male bias in the penetrance of the neurodevelopmental phenotypes at the 16p13.11 locus. Importantly, in re-examining the data reported in the literature, we found a trend toward a male-specific prevalence of 16p13.11 duplications also in schizophrenia-affected patients [19], which provides further support for our findings.

A male biased sex ratio is typical of several neurodevelopmental disorders including attention deficit hyperactivity disorder, Tourette syndrome, dyslexia, language delay and, particularly, autism and Asperger syndrome [52]. The specific factors responsible for the higher male prevalence remain unclear; however, prenatal androgens and, in particular, the exposure to high maternal intrauterine testosterone levels, are thought to play a key role, being putatively responsible for the unusually hypermasculinized traits observed in individuals affected by different male-based neurodevelopmental disorders, such as autism or ADHD [26], [52], [53]. Genetic factors are also likely to be determinant, and recent studies suggest that stronger genetic perturbations are required to trigger an autistic phenotype in females than males, including CNVs encompassing a larger number of genes or CNVs affecting genes that produce a more deleterious impact when disrupted [54], [55]; the specific compensatory mechanisms behind this greater resistance in females are currently unknown, although the well-established sexual differences in cognition, neuroanatomy and neural function observed in males and females across development, are likely to play an important role [26]. A decreased or impaired interhemispheric connectivity, for example, has been associated with different neurodevelompmental disorders, including autism and schizophrenia [56], [57], and interestingly, despite smaller brain size in females than in males, females generally show larger corpora callosa and increased interhemispheric connectivity compared to males [26], which might reflect a higher robustness that offers females protection. Furthermore, another major determinant of the higher threshold of liability observed in females could be represented by dynamic factors that operate above and beyond the generally stable DNA sequence variations, such as sex-specific differences in gene regulation or sexually dimorphic epigenetic processes. Intriguingly, recent findings suggest that the amygdala, a major component of the “social brain”, exhibits sex differences on multiple epigenetic factors during brain development, and specifically, in neonatal males the amygdala shows a decreased expression of both enzymes involved in DNA methylation and factors essential for reading the methylation marks that alter gene expression; this novel observation is thought to reflect an increased window of differentiation for the male amygdala, which could in turn put males at higher risk for developing altered social behaviour and mental health problems later in life, if any perturbation occurs during this extended period of neuronal differentiation [29].

Performing a search for the disease-causing genes in the 16p13.11 region, we observed a significant enrichment of case duplications and deletions containing interval II, which is located between 15.49 and 16.32 Mb and encompasses a core set of nine genes (MPV17L>ABCC6). This particular interval has shown to be exquisitely dosage-sensitive, because pathogenic when either duplicated or deleted and is thus likely to represent the core pathogenic region at the 16p13.11 locus, harbouring the dosage-sensitive genes responsible for the deleterious phenotypes observed in our cases. Finally, we also applied an evolutionary genetic approach and tested for the presence of ohnologs in the 16p13.11 region.

Vertebrate evolution has been characterized by two whole genome duplication (WGD) events; many of the duplicated copies (“ohnologs”) have been lost during the evolution, but some of them have been retained in the vertebrate genome. Over 60% of retained ohnologs seem to be refractory to small-scale duplication (SSD) and copy number variation (CNV) in human populations and are significantly enriched in human disease genes, consequently, they are thought to represent ancient dosage-balanced genes (dosage-balanced ohnologs, DBOs), resistant to copy number change because it leads to deleterious phenotypes under negative genetic selection [42], [58]; ohnologs at the 16p13.11 locus, therefore, may be more likely than other genes to play an important role in the aetiology of the pathological phenotypes associated with the 16p13.11 copy number variants. Consistent with this hypothesis we found that four of the five ohnologs identified at 16p13.11 (NDE1, MYH11, ABCC1 and ABCC6) were contained in the core pathogenic region significantly overrepresented in our case CNVs, and we therefore suggest that they represent important candidates for the observed deleterious phenotypes.

The MYH11 gene encodes the smooth muscle cell (SMC)-specific isoform of β-myosin heavy chain, a major specific contractile protein produced in SMC, and represents the most important candidate gene in the 16p13.11 region for the predisposition to thoracic aortic aneurysm and dissection (TAAD) [25]; heterozygous mutations in the MYH11 gene have been identified in individuals with familial TAAD and are thought to be able to disrupt SMC contractile function, leading to an early and severe decrease in the elasticity of the aortic wall [59].

ABCC1 and ABCC6 encode the multidrug resistance-associated proteins 1 and 6, two members of the ATP-binding cassette (ABC) transporters superfamily [60]. These two genes have not so far been associated with neurodevelopmental conditions; however, mutations in ABCC6 are responsible for pseudoxanthoma elasticum (OMIM 264800), a multisystem genetic disorder characterised by dystrophic mineralisation of soft connective tissues in a number of organs, including skin, eyes and arterial blood vessels [61].

Finally, the NDE1 gene represents the strongest candidate for the neurodevelopmental phenotypes associated with the 16p13.11 CNVs. It encodes the nuclear distribution protein nudE homolog 1, a centrosomal protein which plays a crucial role in the process of mammalian encephalisation and human cerebral cortex growth, because of its involvement in mitosis, neuronal migration and microtubule organization during brain development [62]. Loss of NDE1 in mouse models causes profound defects in cerebral corticogenesis and neuronal proliferation and migration, and mutations in NDE1 have been associated with extreme microlissencephaly in humans [63], [64]. NDE1 and its homolog NDEL1 (Nuclear distribution protein nudE-like 1) physically interact with LIS1 (Lissencephaly 1), the first lissencephaly gene identified, and form a complex involved in neuronal proliferation, differentiation, and migration within the brain [65]. Importantly, the NDE1/LIS1/NDEL1 complex is part of the disrupted in schizophrenia-1 (DISC1) pathway, one of the most relevant pathways underlying psychosis, and directly binds the DISC1 gene; furthermore, NDE1 subcellular localization and protein-protein interactions are modulated through phosphorylation by the cAMP-activated protein kinase A (PKA), and DISC1 is able to modulate the PKA phosphorilation of NDE1 via regulation of the activity of PDE4, a cAMP-hydrolyzing enzyme which creates a co-complex with DISC1 and NDE1/LIS1/NDEL1 [66], [67].

Interestingly, several studies have reported evidence of sex-specific associations of sequence variations in the DISC1 gene and a number of neuropsychiatric disorders, including schizophrenia, bipolar disorder, major depression, autism and Asperger syndrome, as well as with a variety of neurocognitive endophenotypes [68]–[73]. The most robust association has been observed for a two-marker haplotype (HEP3) spanning a 62 kbp genomic region from intron 1 to exon 2 of the DISC1 gene, which was originally found to confer significant risk of schizophrenia only to male probands in a sample of Finnish families [68], and has subsequently been associated to the neurocognitive measure of visual working memory only in males [69]. Notably, a later study in the same sample of Finnish families also found sex-dependent evidence of association between a tag-haplotype in the NDE1 gene and risk of schizophrenia only in females [74].

Further support for a possible sex-specific effect of the DISC1 pathway in conferring risk for mental illness, also comes from animal studies. Using a new transgenic mouse model, Pletnikov et al. found that the expression of a mutant human truncated DISC1 protein (hDISC1) in forebrain areas of the mouse brain produces sex-dependent behavioural effects, with male mice showing increased spontaneous locomotor activity and attenuated social behaviour and female mice exhibiting impaired spatial memory and depression-like behaviour [75], [76]. These evidences suggest the possible involvement of the NDE1 gene and, more generally, of the DISC1 risk pathway, in the sex-biased effect observed for the 16p13.11 copy number variants, however, additional studies will be needed to explore the possible molecular mechanisms mediating such an effect.

Recently, the specific role of NDE1 as disease-causing candidate gene at 16p13.11 has been investigated in a preliminary study by Liu et al. [77], examining surgically resected brain tissues from two patients with mesial temporal lobe epilepsy (MTLE), carrying identical heterozygous 16p13.11 deletions encompassing the NDE1 gene. The authors did not find any detectable structure alteration in the brain tissues with the methods employed, which might suggest that, at least in the two cases examined, the deletion of NDE1 could not represent the key contributor to the patients phenotype, or that it could act at a different level than the one examined in the study [77]; despite the very small clinical sample, the study highlights the importance of deep phenotyping analysis for the interpretation of the present genetic findings, outlining a crucial direction for future work.

There are potential limitations in the case-control analysis performed in the present study, which arise because of the different CNV detection methods used for the generation of the data in cases (Agilent CGH array) and controls (Illumina SNP genotyping arrays). Platform heterogeneity in CNV detection may produce false CNV enrichment signals, as a function of different probe density and distribution (gene-centric probes on array CGH, especially focussed on known disease variants), different analysis methods, etc. This means that not all regions of the genome will be directly comparable, and some CNV identified by array CGH may be missed by Illumina genotyping arrays and vice-versa. Platform heterogeneity, however, is unlikely to represent a major confounding factor in the present study, as platform-specific differences in sensitivity and specificity have been found to largely disappear for large CNVs (>500 kbp in size) [37]–[40], and the 16p13.11 locus is a known CNV region harbouring NAHR-mediated variations with size >800 kbp, and is adequately covered by both our array CGH probes and Illumina genotyping array probes for the detection of such events.

In summary, we studied the impact of duplications and deletions at chromosome 16p13.11 in a clinical sample of children and young adults with a range of neurodevelopmental disorders, analysed by array comparative genomic hybridisation (aCGH). While phenotype-led studies have recently implicated these genomic variations in different specific syndromes [15]–[25], we used a CNV-led approach aiming to capture the full range of phenotypic diversity associated with these pleiotropic variants [12]. The male-biased effect observed in the present study for the 16p13.11 genomic imbalances is intriguing, in that it could help to shed some light on the complex genetic and biological mechanisms underlying the yet poorly understood male bias characteristic of a number of neurodevelopmental disorders. Nearly all human diseases exhibit sexually dimorphic features in prevalence, course and severity; uncovering the bases of such differences could offer unprecedented insights into the aetiology of complex disorders and thus provide concrete means for the design of effective intervention and treatment.

## Supporting Information

Figure S1 **Microdeletions of NTAN1.** Microdeletions at [chr16∶15,131,723–15,154,746] identified in two cases of the referral series (Case 1 and Case 2) and in two cases reported in the DECIPHER database (patients 250140 and 265264) (http://genome.ucsc.edu/).
(TIF)

Figure S2 **NAHR-mediated duplications and deletions of 16p13.11 in the DECIPHER cases.** NAHR-mediated duplications (blue) and deletions (red) identified in the 16p13.11-p12.3 region (Chr16∶14.66–18.70 Mb, GRCh37/hg19) in the DECIPHER referral cases. Segmental duplications in the region are also shown (http://genome.ucsc.edu/).
(TIF)

Table S1 **Genotype–phenotype correlation for patients with duplication or deletion of 16p13.11 identified in the clinical referral series.**
(PDF)

Table S2 **Duplications and deletions of 16p13.11 identified in the control cohorts.**
(PDF)

Table S3 **NAHR-mediated duplications and deletions of 16p13.11 in the DECIPHER cases.**
(PDF)

Table S4 **Genotype–phenotype correlation for patients with duplication or deletion of 16p13.11 recorded in the DECIPHER database.**
(PDF)

Methods S1 **Supplementary methods.**
(PDF)
