# Supplementary figures and images for: Male-Biased Autosomal Effect of 16p13.11 Copy Number Variation in Neurodevelopmental Disorders
Source: PLoS One. 2013 Apr 18;8(4):e61365. doi: 10.1371/journal.pone.0061365 (PMC3630198; doi:10.1371/journal.pone.0061365)

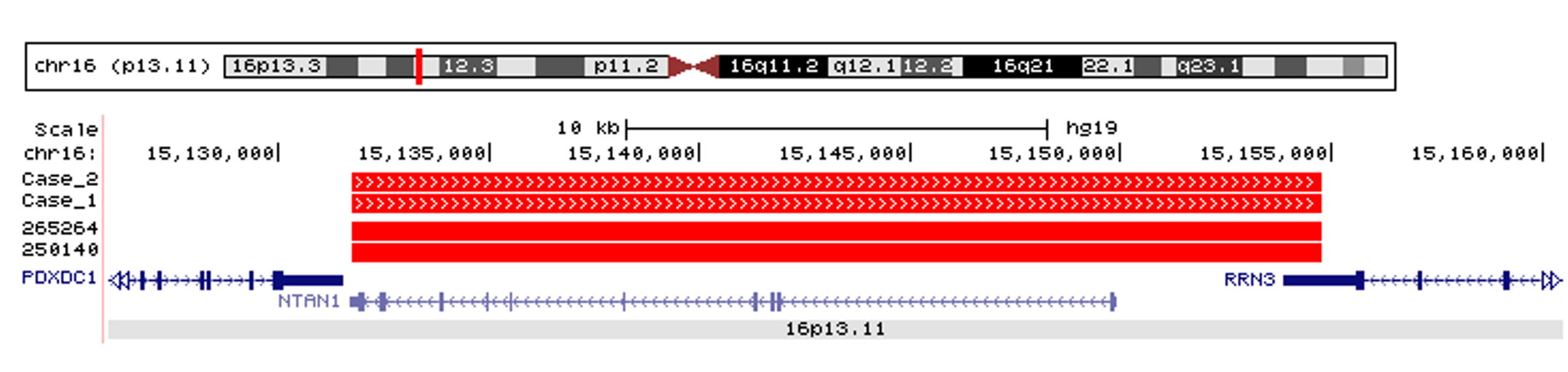

Supplement: Figure S1 — Microdeletions of NTAN1. Microdeletions at [chr16∶15,131,723–15,154,746] identified in two cases of the referral series (Case 1 and Case 2) and in two cases reported in the DECIPHER database (patients 250140 and 265264) (http://genome.ucsc.edu/). (TIF) [file pone.0061365.s001.tif]

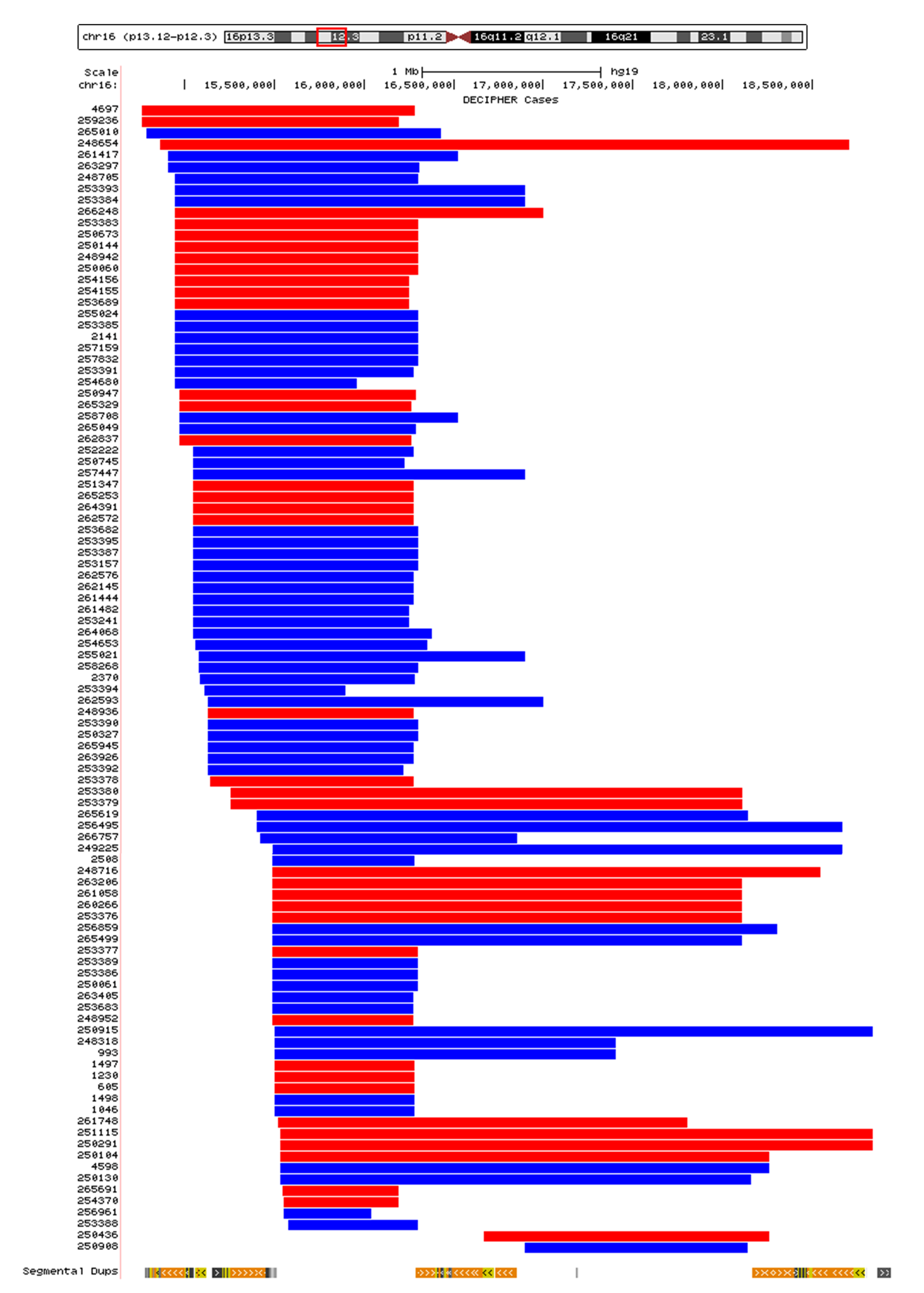

Supplement: Figure S2 — NAHR-mediated duplications and deletions of 16p13.11 in the DECIPHER cases. NAHR-mediated duplications (blue) and deletions (red) identified in the 16p13.11-p12.3 region (Chr16∶14.66–18.70 Mb, GRCh37/hg19) in the DECIPHER referral cases. Segmental duplications in the region are also shown (http://genome.ucsc.edu/). (TIF) [file pone.0061365.s002.tif]
