# Supplementary material for: Male-Biased Autosomal Effect of 16p13.11 Copy Number Variation in Neurodevelopmental Disorders
Source: PLoS One. 2013 Apr 18;8(4):e61365. doi: 10.1371/journal.pone.0061365 (PMC3630198; doi:10.1371/journal.pone.0061365)
Supplement: Table S1 — Genotype–phenotype correlation for patients with duplication or deletion of 16p13.11 identified in the clinical referral series. (PDF) [file pone.0061365.s003.pdf]

Table S1. Genotype–phenotype correlation for patients with duplication or deletion of 16p13.11 identified in the clinical referral series

Case	Diagnosis	Age	Sex	Imbalance count	HG19 start	HG19 stop	Size	Inheritance	CNV	CNV Interval	Genes encompassed by the CNV	Other imbalance
1	Autistic spectrum disorder, learning difficulties	10yrs	M	1	15,131,723	15,154,746	23,024	De novo	x1 ↓	I	NTAN1, RRN3	n/a
2	Attention difficulties, delayed speech and language skills, dysmorphic features	5 yrs	M	2	15,131,723	15,154,746	23,024	De novo	x1 ↓	I	NTAN1, RRN3	**x2 ↑, chrX: 29,488,548-29,973,532 (Maternal)**
3	ASD	7 yrs	M	2	14,780,135	16,305,736	1,525,602	N/K	x3 ↑	I-II	PLA2G10, ABCC6P2, NOMO1, MIR3179-1, MIR3179-2, MIR3179-3, MIR3180-1, MIR3180-2, MIR3180-3, NPIP, PDXDC1, NTAN1, RRN3, MIR3180-4, MPV17L, C16orf45, KIAA0430, NDE1, MIR484, MYH11, FOPNL, ABCC1, ABCC6.	x3 ↑, chr6: 105,548,869-106,951,653 (Maternal)
4	ASD, ventricular septal defect	4 yrs	M	1	14,944,560	16,223,052	1,278,493	Paternal	x3 ↑	I-II	NOMO1, MIR3179-1, MIR3179-2, MIR3179-3, MIR3180-1, MIR3180-2, MIR3180-3, NPIP, PDXDC1, NTAN1, RRN3, MIR3180-4, MPV17L, C16orf45, KIAA0430, NDE1, MIR484, MYH11, FOPNL, ABCC1.	n/a
5	N/K	N/K	M	1	14,944,560	16,249,607	1,305,048	N/K	x3 ↑	I-II	NOMO1, MIR3179-1, MIR3179-2, MIR3179-3, MIR3180-1, MIR3180-2, MIR3180-3, NPIP, PDXDC1, NTAN1, RRN3, MIR3180-4, MPV17L, C16orf45, KIAA0430, NDE1, MIR484, MYH11, FOPNL, ABCC1, ABCC6.	n/a
6	Delayed gross motor skills, motor planning problem, social communication difficulty	3 yrs	F	1	14,944,560	16,276,115	1,331,556	Paternal	x3 ↑	I-II	NOMO1, MIR3179-1, MIR3179-2, MIR3179-3, MIR3180-1, MIR3180-2, MIR3180-3, NPIP, PDXDC1, NTAN1, RRN3, MIR3180-4, MPV17L, C16orf45, KIAA0430, NDE1, MIR484, MYH11, FOPNL, ABCC1, ABCC6.	n/a
7	Absent teeth, bilateral low set ears, chest asymmetry, developmental delay, dysmorphic features., facial asymmetry, gastro-oesophageal reflux, hearing loss, inguinal hernia, inverted left nipple, IUGR/growth failure, lack of hair, metabolic bone disease, plagiocephaly, pseudohydrocephalus, triangular face, undescended testes	5 yrs	M	1	14,944,560	16,305,736	1,361,177	Maternal	x3 ↑	I-II	NOMO1, MIR3179-1, MIR3179-2, MIR3179-3, MIR3180-1, MIR3180-2, MIR3180-3, NPIP, PDXDC1, NTAN1, RRN3, MIR3180-4, MPV17L, C16orf45, KIAA0430, NDE1, MIR484, MYH11, FOPNL, ABCC1, ABCC6.	n/a

8	Global developmental delay	2 yrs	M	1	14,944,560	16,305,736	1,361,177	Paternal	x3 ↑	I-II	NOMO1, MIR3179-1, MIR3179-2, MIR3179-3, MIR3180-1, MIR3180-2, MIR3180-3, NPIP, PDXDC1, NTAN1, RRN3, MIR3180-4, MPV17L, C16orf45, KIAA0430, NDE1, MIR484, MYH11, FOPNL, ABCC1, ABCC6.	n/a
9	DCM, learning difficulties, speech problems	48 yrs	M	1	14,944,560	16,305,736	1,361,177	N/K	x3 ↑	I-II	NOMO1, MIR3179-1, MIR3179-2, MIR3179-3, MIR3180-1, MIR3180-2, MIR3180-3, NPIP, PDXDC1, NTAN1, RRN3, MIR3180-4, MPV17L, C16orf45, KIAA0430, NDE1, MIR484, MYH11, FOPNL, ABCC1, ABCC6.	n/a
10	Arithmetic difficulties, learning difficulties, reading and spelling difficulties, some dysmorphic features	44 yrs	F	1	14,944,560	16,305,736	1,361,177	Paternal	x3 ↑	I-II	NOMO1, MIR3179-1, MIR3179-2, MIR3179-3, MIR3180-1, MIR3180-2, MIR3180-3, NPIP, PDXDC1, NTAN1, RRN3, MIR3180-4, MPV17L, C16orf45, KIAA0430, NDE1, MIR484, MYH11, FOPNL, ABCC1, ABCC6.	n/a
11	Developmental delay, ectopic kidney, microcephaly, renal failure, small for age, UTI	2 yrs	F	2	14,944,560	16,305,736	1,361,177	N/K	x3 ↑	I-II	NOMO1, MIR3179-1, MIR3179-2, MIR3179-3, MIR3180-1, MIR3180-2, MIR3180-3, NPIP, PDXDC1, NTAN1, RRN3, MIR3180-4, MPV17L, C16orf45, KIAA0430, NDE1, MIR484, MYH11, FOPNL, ABCC1, ABCC6.	**x3 ↑, chr22: 21,382,904-24,992,002**
12	ADHD	23 yrs	F	1	14,944,560	16,305,736	1,361,177	N/K	x3 ↑	I-II	NOMO1, MIR3179-1, MIR3179-2, MIR3179-3, MIR3180-1, MIR3180-2, MIR3180-3, NPIP, PDXDC1, NTAN1, RRN3, MIR3180-4, MPV17L, C16orf45, KIAA0430, NDE1, MIR484, MYH11, FOPNL, ABCC1, ABCC6.	n/a
13	Developmental delay, flat nasal bridge, gray matter heterotopia, IUGR, polymicrogyria, prominent forehead	8/12	M	1	14,944,560	16,311,070	1,366,511	Maternal	x3 ↑	I-II	NOMO1, MIR3179-1, MIR3179-2, MIR3179-3, MIR3180-1, MIR3180-2, MIR3180-3, NPIP, PDXDC1, NTAN1, RRN3, MIR3180-4, MPV17L, C16orf45, KIAA0430, NDE1, MIR484, MYH11, FOPNL, ABCC1, ABCC6.	n/a
14	N/K	N/K	M	1	14,944,560	16,311,070	1,366,511	N/K	x3 ↑	I-II	NOMO1, MIR3179-1, MIR3179-2, MIR3179-3, MIR3180-1, MIR3180-2, MIR3180-3, NPIP, PDXDC1, NTAN1, RRN3, MIR3180-4, MPV17L, C16orf45, KIAA0430, NDE1, MIR484, MYH11, FOPNL, ABCC1, ABCC6.	n/a
15	Global developmental delay, functioning at 24-30 months at age 42 months, plagiocephaly, small stature and growth <0.4th	3 yrs	M	1	14,944,560	16,311,070	1,366,511	N/K	x3 ↑	I-II	NOMO1, MIR3179-1, MIR3179-2, MIR3179-3, MIR3180-1, MIR3180-2, MIR3180-3, NPIP, PDXDC1, NTAN1, RRN3, MIR3180-4, MPV17L, C16orf45, KIAA0430, NDE1, MIR484, MYH11, FOPNL, ABCC1, ABCC6.	n/a
16	Global developmental delay, speech delay	3 yrs	M	1	15,048,751	16,276,115	1,227,365	Paternal	x3 ↑	I-II	PDXDC1, NTAN1, RRN3, MIR3180-4, MPV17L, C16orf45, KIAA0430, NDE1, MIR484, MYH11, FOPNL, ABCC1, ABCC6.	n/a
17	N/K	N/K	F	1	15,131,723	16,276,115	1,144,393	Paternal	x3 ↑	I-II	NTAN1, RRN3, MIR3180-4, MPV17L, C16orf45, KIAA0430, NDE1, MIR484, MYH11, FOPNL, ABCC1, ABCC6.	n/a

18	Facial assymetry, learning difficulties, muscular aches - ?myopathic, scoliosis, selective mutism	13 yrs	M	1	15,131,723	16,311,070	1,179,348	De novo	x3 ↑	I-II	NTAN1, RRN3, MIR3180-4, MPV17L, C16orf45, KIAA0430, NDE1, MIR484, MYH11, FOPNL, ABCC1, ABCC6.	n/a
19	Inattentive with immature behaviour, language understanding issues, poor learning pattern, small child	6 yrs	M	1	14,944,560	16,276,115	1,331,556	Maternal	x1 ↓	I-II	NOMO1, MIR3179-1, MIR3179-2, MIR3179-3, MIR3180-1, MIR3180-2, MIR3180-3, NPIP, PDXDC1, NTAN1, RRN3, MIR3180-4, MPV17L, C16orf45, KIAA0430, NDE1, MIR484, MYH11, FOPNL, ABCC1, ABCC6.	n/a
20	Epilepsy, ?ADHD, learning difficulties	27 yrs	M	1	14,944,560	16,276,115	1,331,556	N/K	x1 ↓	I-II	NOMO1, MIR3179-1, MIR3179-2, MIR3179-3, MIR3180-1, MIR3180-2, MIR3180-3, NPIP, PDXDC1, NTAN1, RRN3, MIR3180-4, MPV17L, C16orf45, KIAA0430, NDE1, MIR484, MYH11, FOPNL, ABCC1, ABCC6.	n/a
21	Early onset unusual epilepsy, resistant to treatment, some learning difficulties	15 yrs	M	1	14,944,560	16,305,736	1,361,177	N/K	x1 ↓	I-II	NOMO1, MIR3179-1, MIR3179-2, MIR3179-3, MIR3180-1, MIR3180-2, MIR3180-3, NPIP, PDXDC1, NTAN1, RRN3, MIR3180-4, MPV17L, C16orf45, KIAA0430, NDE1, MIR484, MYH11, FOPNL, ABCC1, ABCC6.	n/a
22	Global developmental delay, very small stature (below the 0.4th centile on all measurements)	2 yrs	M	1	14,944,560	16,305,736	1,361,177	Maternal	x1 ↓	I-II	NOMO1, MIR3179-1, MIR3179-2, MIR3179-3, MIR3180-1, MIR3180-2, MIR3180-3, NPIP, PDXDC1, NTAN1, RRN3, MIR3180-4, MPV17L, C16orf45, KIAA0430, NDE1, MIR484, MYH11, FOPNL, ABCC1, ABCC6.	n/a
23	Bilateral duplication of the hallux, delayed (atypical) cognitive development, digital abnormalities (eg syndactyly, polydactyly), facial dysmorphism (eg hypertelorism), mild delay, umbilical hernia	1 yr	F	1	14,944,560	16,305,736	1,361,177	N/K	x1 ↓	I-II	NOMO1, MIR3179-1, MIR3179-2, MIR3179-3, MIR3180-1, MIR3180-2, MIR3180-3, NPIP, PDXDC1, NTAN1, RRN3, MIR3180-4, MPV17L, C16orf45, KIAA0430, NDE1, MIR484, MYH11, FOPNL, ABCC1, ABCC6.	n/a
24	Social & communication disorder, behavioural difficulties, learning difficulties	12 yrs	M	1	15,048,751	16,276,115	1,227,365	N/K	x1 ↓	I-II	PDXDC1, NTAN1, RRN3, MIR3180-4, MPV17L, C16orf45, KIAA0430, NDE1, MIR484, MYH11, FOPNL, ABCC1, ABCC6.	n/a
25	N/K	N/K	F	1	15,048,751	16,305,736	1,256,986	N/K	x1 ↓	I-II	PDXDC1, NTAN1, RRN3, MIR3180-4, MPV17L, C16orf45, KIAA0430, NDE1, MIR484, MYH11, FOPNL, ABCC1, ABCC6.	n/a
26	Fine motor delay, motor developmental delay, SMA	10/12	M	2	15,131,723	16,249,607	1,117,885	Maternal	x1 ↓	I-II	NTAN1, RRN3, MIR3180-4, MPV17L, C16orf45, KIAA0430, NDE1, MIR484, MYH11, FOPNL, ABCC1, ABCC6.	x1 ↓, chr17: 61,947,138-61,985,739 (Maternal)
27	Autism, very challenging behavior	5 yrs	M	1	15,144,120	16,276,115	1,131,996	Maternal	x1 ↓	I-II	NTAN1, RRN3, MIR3180-4, MPV17L, C16orf45, KIAA0430, NDE1, MIR484, MYH11, FOPNL, ABCC1, ABCC6.	n/a
28	N/K	N/K	N/K	2	14,944,560	18,141,051	3,196,492	Maternal	x3 ↑	I-II-III	NOMO1, MIR3179-1, MIR3179-2, MIR3179-3, MIR3180-1, MIR3180-2, MIR3180-3,	x1 ↓, chr6:

											NPIP, PDXDC1, NTAN1, RRN3, MIR3180-4, MPV17L, C16orf45, KIAA0430, NDE1, MIR484, MYH11, FOPNL, ABCC1, ABCC6, NOMO3, MIR3179-1, MIR3179-2, MIR3179-3, MIR3180-1, MIR3180-2, MIR3180-3, PKD1P1, XYLT1.	26,440,747-26,463,502 (Paternal)
29	Developmental delay, microcephaly	2 yrs	M	2	15,048,751	18,306,854	3,258,104	N/K	x1 ↓	I-II-III	PDXDC1, NTAN1, RRN3, MIR3180-4, MPV17L, C16orf45, KIAA0430, NDE1, MIR484, MYH11, FOPNL, ABCC1, ABCC6, NOMO3, MIR3179-1, MIR3179-2, MIR3179-3, MIR3180-1, MIR3180-2, MIR3180-3, PKD1P1, XYLT1.	**x1 ↓, chr7: 68,564,123-71,234,479**
30	Hypoglycaemia and poor condition at delivery, poor tone and abnormal neurology at 2 months	2/12	M	1	15,496,838	16,311,070	814,233	N/K	x3 ↑	II	MPV17L, C16orf45, KIAA0430, NDE1, MIR484, MYH11, FOPNL, ABCC1, ABCC6.	n/a
31	Hypocalcaemia, slight dysmophic, developmental delay	9/12	M	2	15,256,686	18,141,051	2,884,366	N/K	x3 ↑	II-III	MPV17L, C16orf45, KIAA0430, NDE1, MIR484, MYH11, FOPNL, ABCC1, ABCC6, NOMO3, MIR3179-1, MIR3179-2, MIR3179-3, MIR3180-1, MIR3180-2, MIR3180-3, PKD1P1, XYLT1.	**x3 ↑, chr7: 72,420,745-74,142,327**
32	Developmental delay	3 yrs	M	1	15,256,686	18,141,051	2,884,366	Maternal	x3 ↑	II-III	MPV17L, C16orf45, KIAA0430, NDE1, MIR484, MYH11, FOPNL, ABCC1, ABCC6, NOMO3, MIR3179-1, MIR3179-2, MIR3179-3, MIR3180-1, MIR3180-2, MIR3180-3, PKD1P1, XYLT1.	n/a
33	Asperger's syndrome	4 yrs	M	1	15,256,686	18,141,051	2,884,366	Paternal	x3 ↑	II-III	MPV17L, C16orf45, KIAA0430, NDE1, MIR484, MYH11, FOPNL, ABCC1, ABCC6, NOMO3, MIR3179-1, MIR3179-2, MIR3179-3, MIR3180-1, MIR3180-2, MIR3180-3, PKD1P1, XYLT1.	n/a
34	Complex chromosome 7 rearrangement, developmental delay, dolicocephaly, hypotonia, large tongue, microcephaly, prominent forehead, weight in 50th centile, wide mouth	5 yrs	F	3	15,256,686	18,141,051	2,884,366	N/K	x2~3↑	II-III	MPV17L, C16orf45, KIAA0430, NDE1, MIR484, MYH11, FOPNL, ABCC1, ABCC6, NOMO3, MIR3179-1, MIR3179-2, MIR3179-3, MIR3180-1, MIR3180-2, MIR3180-3, PKD1P1, XYLT1.	**x1~2 ↓, chr7: 100,843,516-122,635,001. x1~2 ↓, chr7: 144,379,972-148,495,065.**
35	Developmental delay, high pain threshold, hypertelorism, poor balance, prominent forehead, small chin + dimple, speech delay	5 yrs	M	1	15,256,686	18,306,854	3,050,169	Maternal (affected)	x3 ↑	II-III	MPV17L, C16orf45, KIAA0430, NDE1, MIR484, MYH11, FOPNL, ABCC1, ABCC6, NOMO3, MIR3179-1, MIR3179-2, MIR3179-3, MIR3180-1, MIR3180-2, MIR3180-3, PKD1P1, XYLT1.	n/a
36	Obesity, raiding cupboards, smallish genitalia, temper tantrums when food is withheld, to exclude	7 yrs	M	1	15,256,686	18,306,854	3,050,169	Maternal	x3 ↑	II-III	MPV17L, C16orf45, KIAA0430, NDE1, MIR484, MYH11, FOPNL, ABCC1, ABCC6, NOMO3, MIR3179-1, MIR3179-2, MIR3179-	n/a

	PWS										3, MIR3180-1, MIR3180-2, MIR3180-3, PKD1P1, XYLT1.	
37	Speech and language delay	5 yrs	M	1	15,256,686	18,546,759	3,290,074	N/K	x3 ↑	II-III	MPV17L, C16orf45, KIAA0430, NDE1, MIR484, MYH11, FOPNL, ABCC1, ABCC6, NOMO3, MIR3179-1, MIR3179-2, MIR3179-3, MIR3180-1, MIR3180-2, MIR3180-3, PKD1P1, XYLT1, MIR3179-1, MIR3179-2, MIR3179-3, MIR3180-1, MIR3180-2, MIR3180-3, NOMO2.	n/a
38	ASD, Asperger's syndrome	9 yrs	M	2	15,492,317	18,141,051	2,648,735	N/K	x3 ↑	II-III	MPV17L, C16orf45, KIAA0430, NDE1, MIR484, MYH11, FOPNL, ABCC1, ABCC6, NOMO3, MIR3179-1, MIR3179-2, MIR3179-3, MIR3180-1, MIR3180-2, MIR3180-3, PKD1P1, XYLT1.	x3 ↑, chrX: 620,267-1,259,140
39	Seizure activity, history of continuous fitting, not dysmorphic	2/12	F	1	15,496,838	18,306,854	2,810,017	N/K	x3 ↑	II-III	MPV17L, C16orf45, KIAA0430, NDE1, MIR484, MYH11, FOPNL, ABCC1, ABCC6, NOMO3, MIR3179-1, MIR3179-2, MIR3179-3, MIR3180-1, MIR3180-2, MIR3180-3, PKD1P1, XYLT1.	n/a
40	Global developmental delay, lissencephaly	17yrs	M	1	15,496,838	18,546,759	3,049,922	Paternal	x3 ↑	II-III	MPV17L, C16orf45, KIAA0430, NDE1, MIR484, MYH11, FOPNL, ABCC1, ABCC6, NOMO3, MIR3179-1, MIR3179-2, MIR3179-3, MIR3180-1, MIR3180-2, MIR3180-3, PKD1P1, XYLT1, MIR3179-1, MIR3179-2, MIR3179-3, MIR3180-1, MIR3180-2, MIR3180-3, NOMO2.	n/a
41	ASD, anomalous tracheobronchial tree, CHD, imperforate anus, IUGR, motor delay, proportionate microcephaly, rectovestibular fistula, very small, ventricular septal defect	10/12	F	2	15,256,686	18,306,854	3,050,169	De novo	x1 ↓	II-III	MPV17L, C16orf45, KIAA0430, NDE1, MIR484, MYH11, FOPNL, ABCC1, ABCC6, NOMO3, MIR3179-1, MIR3179-2, MIR3179-3, MIR3180-1, MIR3180-2, MIR3180-3, PKD1P1, XYLT1.	x1 ↓, chr17: 45,438,799-45,507,261 (Maternal)
42	Raised blood pressure during pregnancy, scalded-skin syndrome	10/12	M	2	15,256,686	18,306,854	3,050,169	Paternal (Affected)	x1 ↓	II-III	MPV17L, C16orf45, KIAA0430, NDE1, MIR484, MYH11, FOPNL, ABCC1, ABCC6, NOMO3, MIR3179-1, MIR3179-2, MIR3179-3, MIR3180-1, MIR3180-2, MIR3180-3, PKD1P1, XYLT1.	x1 ↓, chr13: 23,566,962-24,890,143 (Maternal)
43	Mild learning disabilities, moderate developmental delay, non dysmorphic	5 yrs	M	1	15,492,317	18,306,854	2,814,538	De novo	x1 ↓	II-III	MPV17L, C16orf45, KIAA0430, NDE1, MIR484, MYH11, FOPNL, ABCC1, ABCC6, NOMO3, MIR3179-1, MIR3179-2, MIR3179-3, MIR3180-1, MIR3180-2, MIR3180-3, PKD1P1, XYLT1.	n/a
44	Global developmental delay (severe), motor delay, speech and language delay	4 yrs	M	2	15,492,317	18,306,854	2,814,538	N/K	x1 ↓	II-III	MPV17L, C16orf45, KIAA0430, NDE1, MIR484, MYH11, FOPNL, ABCC1, ABCC6, NOMO3, MIR3179-1, MIR3179-2, MIR3179-3, MIR3180-1, MIR3180-2, MIR3180-3,	x4 ↑, chr4: 70,898,799-70,917,149

											PKD1P1, XYLT1.	
45	Developmental delay	8/12	M	1	15,496,838	18,306,854	2,810,017	Paternal	x1 ↓	II-III	MPV17L, C16orf45, KIAA0430, NDE1, MIR484, MYH11, FOPNL, ABCC1, ABCC6, NOMO3, MIR3179-1, MIR3179-2, MIR3179-3, MIR3180-1, MIR3180-2, MIR3180-3, PKD1P1, XYLT1.	n/a
46	Developmental delay, haemangioma on forehead, prominent forehead, relative microcephaly, short nose, tented upper lip, upturned nose	3 yrs	M	1	15,677,026	18,141,051	2,464,026	Maternal	x1 ↓	II-III	C16orf45, KIAA0430, NDE1, MIR484, MYH11, FOPNL, ABCC1, ABCC6, NOMO3, MIR3179-1, MIR3179-2, MIR3179-3, MIR3180-1, MIR3180-2, MIR3180-3, PKD1P1, XYLT1.	n/a

Abbreviations: ASD, autism spectrum disorder; IUGR, intrauterine growth restriction; DCM, dilated cardiomyopathy; UTI, urinary tract infection; ADHD, attention deficit hyperactivity disorder; SMA, spinal muscular atrophy; PWS, Prader-Willi syndrome; CHD, congenital heart disease; CNV, copy number variant.
Second imbalances in bold are disease-associated.
