# Supplementary material for: Male-Biased Autosomal Effect of 16p13.11 Copy Number Variation in Neurodevelopmental Disorders
Source: PLoS One. 2013 Apr 18;8(4):e61365. doi: 10.1371/journal.pone.0061365 (PMC3630198; doi:10.1371/journal.pone.0061365)
Supplement: Table S2 — Duplications and deletions of 16p13.11 identified in the control cohorts. (PDF) [file pone.0061365.s004.pdf]

Table S2. Duplications and deletions of 16p13.11 identified in the control cohorts

Control	Sex	HG19 start	HG19 stop	Size (bp)	CNV	CNV Interval	Genes encompassed by the CNV	Rare second-hit CNVs >500kbp
WTCCC2_1	F	15,032,942	16,100,661	1,067,719	x3 ↑	I-II	NPIP, PDXDC1, NTAN1, RRN3, MIR3180-4, MPV17L, C16orf45, KIAA0430, NDE1, MIR484, MYH11, FOPNL, ABCC1.	x1 ↓ Chr5: 95,425,820-96,818,211 Size (bp): 1,392,392
WTCCC2_2	F	15,032,942	16,190,572	1,157,630	x3 ↑	I-II	NPIP, PDXDC1, NTAN1, RRN3, MIR3180-4, MPV17L, C16orf45, KIAA0430, NDE1, MIR484, MYH11, FOPNL, ABCC1.	x3 ↑ Chr2: 132,969,856-133,735,535 Size (bp): 765,680
WTCCC2_3	F	15,032,942	16,197,033	1,164,091	x3 ↑	I-II	NPIP, PDXDC1, NTAN1, RRN3, MIR3180-4, MPV17L, C16orf45, KIAA0430, NDE1, MIR484, MYH11, FOPNL, ABCC1.	
WTCCC2_4	M	15,032,942	16,541,073	1,508,132	x3 ↑	I-II	NPIP, PDXDC1, NTAN1, RRN3, MIR3180-4, MPV17L, C16orf45, KIAA0430, NDE1, MIR484, MYH11, FOPNL, ABCC1, ABCC6, NOMO3, MIR3179-1, MIR3179-2, MIR3179-3, MIR3180-1, MIR3180-2, MIR3180-3, PKD1P1.	
Shaikh_1	F	15,032,942	16,541,073	1,508,132	x3 ↑	I-II	NPIP, PDXDC1, NTAN1, RRN3, MIR3180-4, MPV17L, C16orf45, KIAA0430, NDE1, MIR484, MYH11, FOPNL, ABCC1, ABCC6, NOMO3, MIR3179-1, MIR3179-2, MIR3179-3, MIR3180-1, MIR3180-2, MIR3180-3, PKD1P1.	
Shaikh_2	M	15,037,441	16,197,033	1,159,593	x3 ↑	I-II	NPIP, PDXDC1, NTAN1, RRN3, MIR3180-4, MPV17L, C16orf45, KIAA0430, NDE1, MIR484, MYH11, FOPNL, ABCC1.	
WTCCC2_5	F	15,062,399	15,992,737	930,338	x3 ↑	I-II	PDXDC1, NTAN1, RRN3, MIR3180-4, MPV17L, C16orf45, KIAA0430, NDE1, MIR484, MYH11, FOPNL.	
WTCCC2_6	M	15,062,399	16,003,652	941,253	x3 ↑	I-II	PDXDC1, NTAN1, RRN3, MIR3180-4, MPV17L, C16orf45, KIAA0430, NDE1, MIR484, MYH11, FOPNL.	
Cooper_1	F	14,824,194	16,324,781	1,500,587	x1 ↓	I-II	ABCC6P2, NOMO1, MIR3179-1, MIR3179-2, MIR3179-3, MIR3180-1, MIR3180-2, MIR3180-3, NPIP, PDXDC1, NTAN1, RRN3, MIR3180-4,	

							MPV17L, C16orf45, KIAA0430, NDE1, MIR484, MYH11, FOPNL, ABCC1, ABCC6.	
WTCCC2_7	F	15,032,942	16,197,033	1,164,091	x1 ↓	I-II	NPIP, PDXDC1, NTAN1, RRN3, MIR3180-4, MPV17L, C16orf45, KIAA0430, NDE1, MIR484, MYH11, FOPNL, ABCC1.	
WTCCC2_8	F	15,032,942	16,197,033	1,164,091	x1 ↓	I-II	NPIP, PDXDC1, NTAN1, RRN3, MIR3180-4, MPV17L, C16orf45, KIAA0430, NDE1, MIR484, MYH11, FOPNL, ABCC1.	
WTCCC2_9	F	15,032,942	16,197,033	1,164,091	x1 ↓	I-II	NPIP, PDXDC1, NTAN1, RRN3, MIR3180-4, MPV17L, C16orf45, KIAA0430, NDE1, MIR484, MYH11, FOPNL, ABCC1.	
Shaikh_3	F	15,076,261	18,174,650	3,098,390	x3 ↑	I-II-III	PDXDC1, NTAN1, RRN3, MIR3180-4, MPV17L, C16orf45, KIAA0430, NDE1, MIR484, MYH11, FOPNL, ABCC1, ABCC6, NOMO3, MIR3179-1, MIR3179-2, MIR3179-3, MIR3180-1, MIR3180-2, MIR3180-3, PKD1P1, XYLT1.	
Cooper_2	F	15,387,380	18,007,300	2,619,920	x3 ↑	II-III	MPV17L, C16orf45, KIAA0430, NDE1, MIR484, MYH11, FOPNL, ABCC1, ABCC6, NOMO3, MIR3179-1, MIR3179-2, MIR3179-3, MIR3180-1, MIR3180-2, MIR3180-3, PKD1P1, XYLT1.	
WTCCC2_10	F	15,387,380	18,174,650	2,787,270	x3 ↑	II-III	MPV17L, C16orf45, KIAA0430, NDE1, MIR484, MYH11, FOPNL, ABCC1, ABCC6, NOMO3, MIR3179-1, MIR3179-2, MIR3179-3, MIR3180-1, MIR3180-2, MIR3180-3, PKD1P1, XYLT1.	
WTCCC2_11	F	15,387,380	18,174,650	2,787,270	x3 ↑	II-III	MPV17L, C16orf45, KIAA0430, NDE1, MIR484, MYH11, FOPNL, ABCC1, ABCC6, NOMO3, MIR3179-1, MIR3179-2, MIR3179-3, MIR3180-1, MIR3180-2, MIR3180-3, PKD1P1, XYLT1.	
WTCCC2_12	M	15,560,731	17,902,141	2,341,410	x3 ↑	II-III	C16orf45, KIAA0430, NDE1, MIR484, MYH11, FOPNL, ABCC1, ABCC6, NOMO3, MIR3179-1, MIR3179-2, MIR3179-3, MIR3180-1, MIR3180-2, MIR3180-3, PKD1P1, XYLT1.	x1 ↓<br>Chr16:1,110,513- 1,994,156<br>Size (bp): 883,644

Abbreviation: CNV, copy number variant.
