# Supplementary material for: Male-Biased Autosomal Effect of 16p13.11 Copy Number Variation in Neurodevelopmental Disorders
Source: PLoS One. 2013 Apr 18;8(4):e61365. doi: 10.1371/journal.pone.0061365 (PMC3630198; doi:10.1371/journal.pone.0061365)
Supplement: Table S3 — NAHR-mediated duplications and deletions of 16p13.11 in the DECIPHER cases. (PDF) [file pone.0061365.s005.pdf]

Table S3. NAHR-mediated duplications and deletions of 16p13.11 in the DECIPHER cases.

	Total Sample		Male only		Female only	
**16p13.11 CNV**	**Cases (N=18,451)[a]**	**Controls (N=10,375)**	**Cases (N=9,963)**	**Controls (N=4,474)**	**Cases (N=8,488)**	**Controls (N=5,901)**
All Dupl	61	13	32	4	27	9
All Del	40	4	29	0	11	4
Dupl I	0	0	0	0	0	0
Dupl I+II	39	8	22	3	16	5
Dupl I+II+III	0	1	0	0	0	1
Dupl II	11	0	6	0	5	0
Dupl II+III	10	4	4	1	5	3
Dupl III	1	0	0	0	1	0
Del I	0	0	0	0	0	0
Del I+II	20	4	15	0	5	4
Del I+II+III	1	0	1	0	0	0
Del II	7	0	5	0	2	0
Del II+III	11	0	7	0	4	0
Del III	1	0	1	0	0	0

Abbreviations: Dupl, duplication; Del, deletion.
[a] The sex of two cases carrying a duplication of intervals I and II and a duplication of intervals II and III was unknown, consequently, we were unable to include them in the male or female subgroup.
