# Supplementary material for: Male-Biased Autosomal Effect of 16p13.11 Copy Number Variation in Neurodevelopmental Disorders
Source: PLoS One. 2013 Apr 18;8(4):e61365. doi: 10.1371/journal.pone.0061365 (PMC3630198; doi:10.1371/journal.pone.0061365)
Supplement: Table S4 — Genotype–phenotype correlation for patients with duplication or deletion of 16p13.11 recorded in the DECIPHER database. (PDF) [file pone.0061365.s006.pdf]

Table S4. Genotype–phenotype correlation for patients with duplication or deletion of 16p13.11 recorded in the DECIPHER database.

DECIPHER ID	Diagnosis	Age	Sex	Imbalance count	HG19 start	HG19 stop	Size (Mb)	Inheritance	CNV	CNV Interval	Other imbalance
250140	Unknown	Unknown	M	2	15,131,723	15,154,746	0.02	Unknown	x1 ↓	I	x1 ↑, chr2: 110,841,715-110,980,342
265264	Unknown	11 years	F	2	15,131,723	15,154,746	0.02	Unknown	x1 ↓	I	x1 ↓, chr12: 57,997,061-58,025,372
265010	Mental retardation/developmental delay	5 years	M	2	14,784,988	16,432,990	1.65	Familial inherited from parent with similar phenotype to child	x0.58 ↑	I-II	x1 ↓, chr8: 93,045,671-93,317,105
261417	Mental retardation/developmental delay	11 years	other	1	14,910,005	16,525,489	1.62	Unknown	x0.58 ↑	I-II	n/a
263297	Absence of septum pellucidum, duane anomaly, mental retardation/developmental delay, microcephaly, short stature.	2 years	F	2	14,910,227	16,311,040	1.40	Unknown	x0.53 ↑	I-II	**x1 ↓, chr1: 4,658,421-12,912,596**
248705	Cleft uvula, high palate, hypertelorism, hypospadias, mental retardation/developmental delay, palpebral fissures slant down	Unknown	F	2	14,944,559	16,305,736	1.36	Familial inherited from normal parent	x0.52 ↑	I-II	**x0.55 ↓, chr2: 241,168,044-242,102,761**
254680	Psychomotor delay, stereotypic movements, behavioural problems	3 years	M	1	14,944,560	15,960,084	1.02	Familial inherited from normal mother	x0.58 ↑	I-II	n/a
253391	Mental retardation/ developmental delay, speech delay, learning difficulties, obesity	46 years	M	1	14,944,560	16,276,115	1.33	Familial inherited from mildly affected mother (MR/DD)	x 0.6 ↑	I-II	n/a
257832	Severe intellectual disability, abnormal behaviour	16 years	F	1	14,944,560	16,305,677	1.36	Familial inherited from mildly affected father (speech delay)	x0.58 ↑	I-II	n/a
257159	Mental retardation/ developmental delay, speech delay, learning difficulties, motor delay	7 years	F	1	14,944,560	16,305,716	1.36	Familial inherited from mildly affected mother (MR/DD)	x0.58 ↑	I-II	n/a
2141	Advanced bone age/large epiphyses, autism/autistic behavior, generalized obesity, high birth weight (> 90th centile), kidneys general abnormalities, mental retardation/developmental delay, sleep disorders excl.	11 years	M	1	14,944,560	16,305,736	1.36	Unknown	x0.6 ↑	I-II	n/a

	apnoea, tall stature, proportionate										
253385	Mental retardation/ developmental delay, multiple congenital anomalies	1 year	M	2	14,944,560	16,305,736	1.36	De novo	x 0.6 ↑	I-II	x1 ↑, chrX: 139,585,260-139,743,254
255024	Mental retardation/ developmental delay, autism spectrum disorder, speech delay, learning difficulties, motor delay	6 years	M	1	14,944,560	16,305,736	1.36	Familial inherited from mildly affected mother (MR/DD)	x0.58 ↑	I-II	n/a
253384	Mental retardation/ developmental delay, speech delay, learning difficulties, physical dysmorphism, motor delay	13 years	M	1	14,944,560	16,900,216	1.96	Familial inherited from normal mother	x 0.6 ↑	I-II	n/a
253393	Mental retardation/ developmental delay, ADHD, speech delay, learning difficulties, macrocephaly, physical dysmorphism, motor delay	7 years	M	1	14,944,560	16,900,216	1.96	Familial inherited from mildly affected mother (MR/DD)	x0.6 ↑	I-II	n/a
265049	Mental retardation/developmental delay, seizure	17 years	F	2	14,968,855	16,292,235	1.32	Familial inherited from normal mother	x0.58 ↑	I-II	x0.58 ↑, chr10: 92,095,432-92,594,891
258708	Infantile spasms, mental retardation/developmental delay	1 year	F	1	14,968,855	16,525,348	1.56	Unknown	x0.58 ↑	I-II	n/a
252222	Global psychomotor delay, severe speech delay, deafness, behavioural problems, brachycephaly, triangular and flat face, ptosis, high arched eyebrows, hypertelorism, short nares.	4 years	M	2	15,048,551	16,276,256	1.23	Familial inherited from normal mother	x0.48 ↑	I-II	x0.61↑, chr11: 10,847,816-11,588,979
250745	Autism/autistic behavior, mental retardation/developmental delay	3 years	M	1	15,048,660	16,227,943	1.18	Familial inherited from normal mother	x0.5 ↑	I-II	n/a
253241	Mental retardation/ developmental delay, speech delay, learning difficulties, multiple congenital anomalies, physical dysmorphism, motor delay	6 years	F	3	15,048,751	16,249,607	1.20	Familial inherited from normal mother	x0.6 ↑	I-II	x1 ↓, chr11: 76,376,798-85,689,145. x0.6↑, chr1: 160,797,550-161,275,220
261482	Unknown	Unknown	F	1	15,048,751	16,249,607	1.20	Unknown	x0.58 ↑	I-II	n/a
261444	Mental retardation/ developmental delay, learning difficulties	11 years	M	1	15,048,751	16,276,115	1.23	Familial inherited from mildly affected father (MR/DD)	x0.58 ↑	I-II	n/a
262145	Mental retardation/ developmental delay, learning difficulties	19 years	M	1	15,048,751	16,276,115	1.23	Familial inherited from normal father	x0.58 ↑	I-II	n/a
262576	Unknown	21 years	F	1	15,048,751	16,276,115	1.23	Unknown	x0.58 ↑	I-II	n/a

253157	Mental retardation/ developmental delay, speech delay, learning difficulties, physical dysmorphism	9 years	F	2	15,048,751	16,305,736	1.26	Familial inherited from severely affected mother with similar phenotype to child	x0.6↑	I-II	x0.6 ↑, chr4: 113,930,347-114,469,901
253387	Unknown	1 year	F	1	15,048,751	16,305,736	1.26	Unknown	x0.6 ↑	I-II	n/a
253395	Mental retardation/ developmental delay, speech delay, learning difficulties, physical dysmorphism	8 years	M	1	15,048,751	16,305,736	1.26	Familial inherited from mildly affected mother (MR/DD)	x0.6 ↑	I-II	n/a
253682	General facial abnormalities, mental retardation/developmental delay, seizures, general neurological abnormalities	5 years	F	1	15,048,751	16,305,736	1.26	Familial inherited from normal mother	x0.63 ↑	I-II	n/a
257447	Mental retardation/ developmental delay, speech delay, learning difficulties, behavioural problems, motor delay	10 years	M	2	15,048,751	16,899,676	1.85	Familial inherited from mildly affected mother (MR/DD)	x0.58 ↑	I-II	x1 ↓, chr2: 136,103,113-136,226,458
264068	Unknown	5 years	M	2	15,049,872	16,377,650	1.33	Familial inherited from normal father	x0.58 ↑	I-II	x0.58 ↑, chrX: 148,739,101-148,839,812
254653	Unknown	8 years	F	1	15,061,354	16,351,783	1.29	Familial inherited from normal parent	x0.58 ↑	I-II	n/a
258268	Unknown	9 years	F	1	15,081,354	16,305,736	1.22	Unknown	x0.58 ↑	I-II	n/a
255021	Mental retardation/ developmental delay, speech delay, learning difficulties, obesity, motor delay	7 years	F	1	15,081,354	16,899,676	1.82	Familial inherited from mildly affected father (MR/DD)	x0.58 ↑	I-II	n/a
2370	Clinodactyly, flat face, generalized obesity, kyphosis, mental retardation/developmental delay, microstomia, patchy depigmentation of hair/white forelock, scoliosis, short attention span, speech delay	13 years	M	1	15,085,753	16,282,307	1.20	De novo	x3 ↑	I-II	n/a
253394	Mental retardation/ developmental delay, speech delay, learning difficulties, microcephaly	6 years	M	1	15,111,247	15,896,016	0.78	Familial inherited from mildly affected father (MR/DD)	x0.6↑	I-II	n/a
253392	Unknown	19 years	M	2	15,131,723	16,223,052	1.09	Unknown	x0.6 ↑	I-II	x0.6 ↑, chr8: 120,743,057-120,942,109
263926	Mental retardation/ developmental delay, speech delay, learning difficulties, multiple congenital	16 years	F	1	15,131,723	16,276,115	1.14	Familial inherited from normal mother	x0.58 ↑	I-II	n/a

	anomalies, physical dysmorphism, motor delay										
265945	Mental retardation/ developmental delay, ADHD, speech delay, learning difficulties, physical dysmorphism	6 years	M	1	15,131,723	16,276,115	1.14	Familial inherited from mildly affected mother (MR/DD)	x0.58 ↑	I-II	n/a
250327	Mental retardation/developmental delay	Unknown	M	2	15,131,723	16,305,736	1.17	Familial inherited from normal father	x0.56 ↑	I-II	x1 ↓, chr2: 192,543,344-199,484,582
253390	Mental retardation/ developmental delay, ADHD, learning difficulties	6 years	M	1	15,131,723	16,305,736	1.17	Familial inherited from mildly affected father (MR/DD)	x0.6 ↑	I-II	n/a
262593	Mental retardation/ developmental delay, speech delay, multiple congenital anomalies, physical dysmorphism, motor delay	2 years	M	3	15,131,723	16,999,359	1.87	Familial inherited from mildly affected father (MR/DD)	x0.58 ↑	I-II	x0.58 ↑, chrX: 73,505,754-74,131,641. **x1 ↓, chr15: 20,481,702-23,179,948**
4697	Camptodactyly, high palate, high/prominent nasal bridge, mental retardation/developmental delay, microcephaly, seizures, general neurological abnormalities, short palpebral fissures, simple ears, syndactyly 2-3 of toes	17 years	M	1	14,759,767	16,282,307	1.52	Unknown	x1 ↓	I-II	n/a
259236	Flat malar region, mental retardation/developmental delay, palpebral fissures slant down, simple/absent philtrum, tonic/clonic (grand-mal)	13 years	M	2	14,762,269	16,194,548	1.43	Familial inherited from normal mother	x1 ↓	I-II	x0.58 ↑, chr3: 141,810,136-142,180,905
253689	Mental retardation/developmental delay	Unknown	M	2	14,944,560	16,249,607	1.31	Familial inherited from normal father	x0.62 ↓	I-II	x0.58 ↑, chr19: 5,155,515-5,641,847
254155	Unknown	14 years	M	1	14,944,560	16,249,607	1.31	Unknown	x1 ↓	I-II	n/a
254156	Mental retardation/ developmental delay, epilepsy, speech delay, learning difficulties, multiple congenital anomalies, physical dysmorphism, motor delay	18 years	F	1	14,944,560	16,249,607	1.31	Familial inherited from mildly affected mother (MR/DD)	x1 ↓	I-II	n/a
250060	Bladder abnormalities, mental retardation/developmental delay, post-axial polydactyly of fingers, seizures	Unknown	M	1	14,944,560	16,305,736	1.36	Familial inherited from normal mother	x0.7 ↓	I-II	n/a
248942	Mental retardation/developmental	8 years	M	1	14,944,560	16,305,736	1.36	De novo	x1 ↓	I-II	n/a

	delay, microcephaly										
250144	Unknown	Unknown	F	1	14,944,560	16,305,736	1.36	Unknown	x1 ↓	I-II	n/a
250673	Unknown	Unknown	F	1	14,944,560	16,305,736	1.36	Unknown	x1 ↓	I-II	n/a
253383	Mental retardation/ developmental delay, epilepsy, speech delay, microcephaly, motor delay	2 years	F	1	14,944,560	16,305,736	1.36	Familial inherited from mildly affected father (MR/DD)	x1 ↓	I-II	n/a
266248	Arched eyebrows, broad nasal tip, mental retardation /developmental delay, prominent mandible, short neck, synophrys.	1 years	M	3	14,944,560	16,999,359	2.05	De novo	x1 ↓	I-II	**x1 ↓, chr1: 244,571,975-246,704,522.** x1 ↓, chr3: 179,066,692-179,161,826
265329	Face abnormalities, mental retardation/developmental delay.	10 years	M	1	14,968,855	16,267,306	1.30	Familial inherited from normal father	x1 ↓	I-II	n/a
250947	Nuchal bleb/cystic hygroma of neck	Unknown	M	1	14,968,855	16,292,235	1.32	De novo	x0.95 ↓	I-II	n/a
262837	Mental retardation/developmental delay, seizure, speech delay	21 years	F	1	14,968,856	16,267,250	1.30	Unknown	x1 ↓	I-II	n/a
262572	Unknown	11 years	M	1	15,048,751	16,276,115	1.23	Unknown	x1 ↓	I-II	n/a
264391	Unknown	5 years	M	2	15,048,751	16,276,115	1.23	Unknown	x1 ↓	I-II	x0.58 ↑, chr1: 20,830,940-21,076,211
265253	Unknown	3 years	M	1	15,048,751	16,276,115	1.23	Unknown	x1 ↓	I-II	n/a
251347	Autism/autistic behaviour	8 years	M	1	15,048,751	16,276,115	1.23	Unknown	x1 ↓	I-II	n/a
248936	Club foot, varus, mental retardation/developmental delay	6 years	M	1	15,131,723	16,276,115	1.14	Unknown	x1 ↓	I-II	n/a
253378	Mental retardation/ developmental delay, autism spectrum disorder, motor delay	2 years	M	1	15,144,120	16,276,115	1.13	Familial inherited from mildly affected father (MR/DD)	x1 ↓	I-II	n/a
248654	Almond shaped palpebral fissures, columella below alae nasi, cryptorchid testes, face general abnormalities, inguinal hernia, kyphosis, large ears, long philtrum, macrocephaly, mental retardation/developmental delay, myopia, nevi or lentigines, obesity, general build abnormalities, premature greying of hair, round face, thin lower lip, thin upper lip	27 years	M	31	14,864,502	18,707,663	3.84	Unknown	x0.79↓	I-II-III	See table legend
266757	Unknown	4 years	M	2	15,423,703	16,855,348	1.43	Familial inherited	x0.58↑	II	x1 ↓,

								from normal father			chr4: 177,167,513-182,264,756
2508	Arachnodactyly, autism/autistic behavior, feeding problems in infants, fine hair, mental retardation/developmental delay, short stature, strabismus, thin or slender build	16 years	F	1	15,491,527	16,282,387	0.79	Familial inherited from normal mother	x0.6↑	II	n/a
253683	General facial abnormalities, mental retardation/developmental delay	Unknown	M	1	15,492,317	16,276,115	0.78	Familial inherited from normal mother	x0.61 ↑	II	n/a
263405	Unknown	7 years	F	2	15,492,317	16,276,175	0.78	Unknown	x0.58 ↑	II	x1 ↑ chr16: 16,899,617-28,574,419
250061	Congenital cardiac anomaly	Unknown	M	1	15,492,317	16,305,736	0.81	De novo	x0.5 ↑	II	n/a
253386	Mental retardation/ developmental delay, ADHD, speech delay, learning difficulties, obesity, motor delay	26 years	F	1	15,492,317	16,305,736	0.81	Familial inherited from mildly affected mother (MR/DD)	x0.6 ↑	II	n/a
253389	Mental retardation/ developmental delay, speech delay, behavioural problems, physical dysmorphism	2 years	M	1	15,492,317	16,305,736	0.81	Familial inherited from mildly affected mother (MR/DD)	x0.6 ↑	II	n/a
1046	Behavioral problems, general neurological abnormalities, constipation, headache/migraine/body pain, megacolon or Hirschsprung syndrome, mental retardation/developmental delay	17 years	M	1	15,504,454	16,284,248	0.78	Unknown	x0.52 ↑	II	n/a
1498	Cleft palate, mental retardation/developmental delay, small mandible/micrognathia, triphalangeal thumb	8 years	F	1	15,504,454	16,284,248	0.78	Unknown	x0.6 ↑	II	n/a
256961	Mental retardation/developmental delay	6 years	M	1	15,551,331	16,041,568	0.49	Familial inherited from normal mother	x0.85 ↑	II	n/a
253388	Mental retardation/ developmental delay, speech delay, learning difficulties, microcephaly, motor delay	8 years	F	1	15,579,295	16,305,736	0.73	Familial inherited from normal mother	x0.6↑	II	n/a
253377	Mental retardation/ developmental delay, speech delay, learning difficulties, microcephaly, physical dysmorphism, motor delay	7 years	F	1	15,492,317	16,305,736	0.81	Familial inherited from mildly affected mother (MR/DD)	x1 ↓	II	n/a
248952	Unknown	Unknown	M	1	15,492,345	16,276,086	0.78	De novo	x1 ↓	II	n/a
605	Cataract, mental	13 years	M	1	15,504,454	16,284,248	0.78	Familial inherited	x0.78 ↓	II	n/a

	retardation/developmental delay							from normal mother			
1230	Auricular tags, cleft palate, complete absence of nose, cyclopia, dysplastic ears, holoprosencephaly/arhinencephaly, midline cleft upper lip, narrow/atretic auditory canal	Unknown	F	1	15,504,454	16,284,248	0.78	Familial inherited from normal parent	x0.86 ↓	II	n/a
1497	Constipation, hallux valgus, macrocephaly, mental retardation/developmental delay	18 years	M	1	15,504,454	16,284,248	0.78	Familial inherited from normal parent	x0.81 ↓	II	n/a
265691	Unknown	1 year	M	1	15,551,102	16,194,719	0.64	Familial inherited from normal father	x1 ↓	II	n/a
254370	Unknown	Unknown	M	1	15,551,302	16,194,578	0.64	Unknown	x1 ↓	II	n/a
265619	Unknown	29 years	M	1	15,404,481	18,141,021	2.74	Unknown	x0.58 ↑	II-III	n/a
256495	Unknown	Unknown	M	2	15,404,482	18,669,696	3.27	Familial inherited from normal parent	x0.5 ↑	II-III	x0.5 ↓, chr10: 96,540,466-98,634,100
249225	Agenesis/hypoplasia of corpus callosum, club foot, varus, glabella bone defect, high/prominent nasal bridge, polydactyly/bifid hallux, short stature, short limbs, small/hypoplastic/deepset nails	Unknown	M	1	15,491,527	18,669,725	3.18	Familial inherited from normal mother	x0.6 ↑	II-III	n/a
265499	Mental retardation/ developmental delay, ADHD, speech delay, learning difficulties, multiple congenital anomalies, physical dysmorphism, obesity, motor delay	6 years	M	1	15,492,317	18,112,776	2.62	Familial inherited from mildly affected father (MR/DD)	x0.58 ↑	II-III	n/a
256859	Mental retardation/developmental delay, round face	2 years	Unknown	3	15,492,317	18,306,854	2.81	De novo	x0.58 ↑	II-III	**x0.58↑, chr15: 20,239,986-30,730,572** x0.58 ↑, chr15: 20,481,702-22,558,756
993	Asymmetric face, atrial septum defect, choanal atresia/stenosis, coloboma of retina/choroid, dilated ureters/ureteral atresia, dysplastic ears, epicanthic folds, myopia, N.VII abnormal/palsy (nerve only), primary amenorrhea, renal agenesis, short neck, simple ears, strabismus,	21 years	F	1	15,504,454	17,409,257	1.90	Familial inherited from normal parent	x0.51 ↑	II-III	n/a

	vestibular apparatus abnormalities										
248318	Autism/autistic behavior, facial abnormalities, mental retardation/developmental delay, speech delay, tall stature	12 years	F	1	15,504,454	17,409,257	1.90	Unknown	x0.35 ↑	II-III	n/a
250915	Autism/autistic behavior, facial abnormalities, hypotonia (non-myopathic), low-set ears, mental retardation/developmental delay, microcephaly, mid-face hypoplasia, short stature, proportionate, small feet, small hands	12 years	F	3	15,504,454	18,839,990	3.34	Familial inherited from normal mother	x0.5 ↑	II-III	**x0.79↓, chr1: 241,469,419-248,865,374** x0.4 ↑, chr20: 61,267,634-62,906,195
250130	Strabismus/gaze palsy, mental retardation/developmental delay	3 years	F	1	15,533,023	18,164,698	2.63	De novo	x0.34 ↑	II-III	n/a
4598	Abnormal sacrum, absent ribs, atrial septum defect, auricular pits/fistulas, clinodactyly, club foot, varus, deep-set eyes, hemivertebrae, hypoplastic or absent tibia, narrow thorax/funnel chest, polydactyly/bifid thumb, scoliosis, trachea or laryngeal anomalies	Unknown	F	1	15,533,023	18,264,088	2.73	Unknown	x0.58 ↑	II-III	n/a
253379	Mental retardation/ developmental delay, motor delay	Unknown	M	1	15,256,686	18,112,776	2.86	Familial inherited from normal father	x1 ↓	II-III	n/a
253380	Mental retardation/ developmental delay, speech delay, learning difficulties, microcephaly, physical dysmorphism, motor delay	4 years	F	1	15,256,686	18,112,776	2.86	Familial inherited from mildly affected mother (MR/DD)	x1 ↓	II-III	n/a
248716	Paroxysmal apnoea/tachypnoea, cerebellar hemisphere hypoplasia, feeding problems in infants, large (mega) cisterna magna, mental retardation/developmental delay, post-axial polydactyly of toes	Unknown	F	2	15,492,316	18,546,759	3.05	Familial inherited from normal parent	x0.86 ↓	II-III	**x1 ↓, chr3: 1,213,906-2,218,784**
253376	Mental retardation/ developmental delay, speech delay, motor delay	2 years	M	1	15,492,317	18,112,776	2.62	Familial inherited from mildly affected father (MR/DD)	x1 ↓	II-III	n/a
260266	Unknown	18 years	M	1	15,492,317	18,112,776	2.62	Unknown	x1 ↓	II-III	n/a
261058	Mental retardation/ developmental delay, speech delay, learning difficulties, motor delay	Unknown	M	2	15,492,317	18,112,776	2.62	Familial inherited from mildly affected father (MR/DD)	x1 ↓	II-III	**x0.58 ↑, chr1: 144,995,225-147,786,706**

263206	Mental retardation/ developmental delay, autism spectrum disorder, speech delay, learning difficulties, motor delay	6 years	M	1	15,492,317	18,112,776	2.62	De novo	x0,5 ↓	II-III	n/a
261748	Cleft palate, mental retardation/developmental delay, narrow thorax/funnel chest, patchy pigment of skin/cafe au lait spots, pulmonary stenosis, short stature, small mandible/micrognathia	12 years	F	2	15,521,713	17,804,366	2.28	De novo	x1 ↓	II-III	**x1 ↓, chr15: 31,728,919-39,743,689**
250104	Behavioral problems, fetal finger pads, mental retardation/developmental delay	Unknown	M	1	15,533,023	18,264,088	2.73	De novo	x1 ↓	II-III	n/a
250291	Unknown	Unknown	M	1	15,533,023	18,839,788	3.31	Familial inherited from normal parent	x1 ↓	II-III	n/a
251115	Mental retardation/developmental delay, seizures, Vision general abnormalities	15 years	F	1	15,533,023	18,839,788	3.31	Familial inherited from normal parent	x1 ↓	II-III	n/a
250908	Unknown	Unknown	F	3	16,899,617	18,141,051	1.24	Unknown	x0.5 ↑	III	x0.5 ↑, chr6: 142,233,257-143,089,666 **x0.8 ↓, chr22: 18,894,835-21,505,417**
250436	Short stature, general abnormalities	Unknown	M	1	16,668,903	18,264,088	1.60	Unknown	x1 ↓	III	n/a

Abbreviation: CNV, copy number variant.
Second imbalances in bold are disease-associated.

Patient 248654, additional genomic imbalances:
Chr1: 104163703-104297301 x0.73↑, Chr2: 89122523-89475400 x0.51↑, Chr3: 195315926-195732015 x0.47↓, Chr4: 9324656-9359762 x1.44↑, Chr4: 10212244-10227435 x4.59↑, Chr4: 34781004-34814779 x1.44↑, Chr4: 69386967-69500230 x3.18↑, Chr4: 69456441-69483277 x5.9↑, Chr4: 94059618-94104576 x1.23↑, Chr4: 168810788-168991830 x0.95↑, Chr5 12813036-12820111 x6.22↓, Chr5: 140223255-140227460 x5.57↑, Chr6: 103738389-103753939 x5.16↑, Chr7: 141730168-141785258 x0.78↓, Chr10: 46756764-48372528 x0.44↓, Chr11: 5792681-5805665 x5.27↑, Chr12: 9637322-9693948 x1.60↑, Chr13: 70747355-70762514 x1.12↑, Chr14: 19434574-19554442 x0.59↓, Chr14: 106263099-106811243 x0.76↑, Chr14: 106334906-106351204 x5.07↑, Chr15: 24487786-24504519 x2.54↑, Chr15: 34695165-34841446 x0.61↑, Chr16: 34452584-34747588 x0.48↓, Chr16: 74375793-74442234 x0.92↓, Chr18: 65855190-65891899 x0.77↓, Chr22: 20326985-20465445 x0.72↓, Chr22: 22462628-23238919 x0.37↑, Chr22: 24376165-24395353 x3.11↑, ChrX: 70121-160747 x1.75↑.
