# Supplementary material for: Male-Biased Autosomal Effect of 16p13.11 Copy Number Variation in Neurodevelopmental Disorders
Source: PLoS One. 2013 Apr 18;8(4):e61365. doi: 10.1371/journal.pone.0061365 (PMC3630198; doi:10.1371/journal.pone.0061365)
Supplement: Methods S1 — Supplementary methods. (PDF) [file pone.0061365.s007.pdf]

Supplementary Methods

Male-biased autosomal effect of 16p13.11 copy number variation in neurodevelopmental disorders

Maria Tropeano[1]*, Joo Wook Ahn[2], Richard JB Dobson[1], Gerome Breen[1], James Rucker[1], Abhishek Dixit[1], Deb K Pal[3], Peter McGuffin[1], Anne Farmer[1], Peter S White[4,5], Joris Andrieux[6], Evangelos Vassos[1], Caroline Mackie Ogilvie[2], Sarah Curran[1], David A Collier[1,7]*

(1) MRC Social, Genetic and Developmental Psychiatry Centre, Institute of Psychiatry, King's College London, London, UK.
(2) Department of Cytogenetics, Guy's and St Thomas' NHS Foundation Trust, London, UK.
(3) Department of Clinical Neuroscience, Institute of Psychiatry, King's College London, London, UK.
(4) Center for Biomedical Informatics, The Children's Hospital of Philadelphia, Philadelphia, Pennsylvania, USA.
(5) Division of Oncology, The Children's Hospital of Philadelphia, Philadelphia, Pennsylvania, USA.
(6) Institut de Génétique Médicale, CHRU de Lille, Lille, France.
(7) Discovery Neuroscience Research, Eli Lilly and Company Ltd, Lilly Research Laboratories, Erl Wood Manor, Windlesham, Surrey, UK.

Correspondence: maria.tropeano@kcl.ac.uk ; collier_david_andrew@lilly.com .

Control CNV analysis and quality controls

Screened control samples were genotyped on the Illumina (San Diego, California, USA) HumanHap 610-Quad beadchip, whereas WTCCC2 samples were genotyped on a modified Illumina 1M beadchip. To ensure comparable CNV detection from different array types, control CNVs were identified using a consensus marker set between the 610K and 1M chips, which mimics the Illumina HumanHap 550 beadchip. We were granted access to the chip intensity data (Illumina '.idat' files). All probe intensity data were normalised and processed using standard Illumina protocols with Illumina's GenomeStudio platform to obtain the log R ratio (LRR) and B allele frequency (BAF) at each marker. The LRR and BAF represent, for each marker in each sample, a summed probe intensity ratio derived from comparison to a canonical value calculated from all samples and, in the case of bi-allelic probes, an allelic intensity ratio respectively. We processed LRR and BAF values for 562,680 autosomal markers (99.9% SNP markers) using PennCNV (version released August 2009), a popular, open-source package designed for Illumina array data that implements a hidden Markov model, a Viterbi algorithm, expectation maximisation and takes account of the distance between consecutive markers to make copy number calls.

Samples with a genotype call rate of less than 98%, a B allele frequency standard deviation (BAFSD) of more than 0.045 or a log R ratio standard deviation (LRRSD) of more than 0.3 were excluded from analysis. We excluded all CNVs of less than 100kb and all CNVs made with less than 10 consecutive markers. We excluded samples with more than a total of 20 CNVs, as such samples are more likely to contain artefactual calls. We excluded calls that fell within 500kb of the centromere or telomere of each chromosome and any calls falling within immunoglobulin regions, as such calls are more likely in DNA derived from immortalised cell lines. We excluded calls occurring in more than 1% of the sample, and calls made over regions of the genome where the marker density was low (<1 marker per 200,000bp).

Table SM1a. Samples surviving each stage of quality control. Stage 1- exclusion by genotype call rate (<98%). Stage 2- exclusion by LRRSD (≤0.3) BAFSD (≤0.045) across autosomal chromosomes and total number of CNVs (≤20) across autosomal chromosomes. NB. A larger proportion of screened control samples were excluded at QC stage 1due to a batch of samples with poor DNA quality.

<table>
<tr><th rowspan="2">QC stage</th><th rowspan="2">Gender</th><th rowspan="2">Screened controls</th><th colspan="2">WTCCC2 controls</th></tr>
<tr><th>NBS</th><th>58C</th></tr>
<tr><td rowspan="4">All samples</td><td>Male</td><td>100.00%</td><td>100.00%</td><td>100.00%</td></tr>
<tr><td>Female</td><td>100.00%</td><td>100.00%</td><td>100.00%</td></tr>
<tr><td rowspan="2">Total</td><td rowspan="2">100.00%</td><td>100.00%</td><td>100.00%</td></tr>
<tr><td colspan="2">100.00%</td></tr>
<tr><td rowspan="4">Post QC stage 1</td><td>Male</td><td>87.08%</td><td>95.46%</td><td>94.93%</td></tr>
<tr><td>Female</td><td>95.73%</td><td>96.82%</td><td>95.85%</td></tr>
<tr><td rowspan="2">Total</td><td rowspan="2">92.37%</td><td>96.15%</td><td>95.38%</td></tr>
<tr><td colspan="2">95.75%</td></tr>
<tr><td rowspan="4">Post QC stage 2</td><td>Male</td><td>74.19%</td><td>90.50%</td><td>87.90%</td></tr>
<tr><td>Female</td><td>86.62%</td><td>90.16%</td><td>90.54%</td></tr>
<tr><td rowspan="2">Total</td><td rowspan="2">82.08%</td><td>90.33%</td><td>89.19%</td></tr>
<tr><td colspan="2">89.74%</td></tr>
</table>

Table SM1b. CNV quality control. QC stage 1- any CNV with a constituent number of SNPs of less than 10 or a length less than 100kb was removed. QC stage 2- all CNVs with more than 50% overlap with immunoglobulin regions or regions defined as within 500kb of the centromere or telomere (Table SM1c) were removed. QC stage 3- all samples with more than a total of 20 CNVs were removed. QC stage 4- all CNVs from genomic regions with less than 1 marker per 200kb in our marker set were removed. QC stage 5- all CNVs occurring at >1% frequency in our population control sample were removed from all cohorts. NB The relatively large proportion of calls remaining in the WTCCC2 sample at QC stages 1 and 2 are likely to be attributed to variants introduced during cell line creation in the 1958 birth cohort.

Cohort/QC Stage	Screened Controls	WTCCC2 Controls
**All CNVs (post sample QC)**	5,710	120,042
	100.00%	100.00%
**QC stage 1**	736	34,408
	12.89%	28.66%
**QC stage 2**	676	33,133
	11.84%	27.60%
**QC stage 3**	507	9,288
	8.88%	7.74%
**QC stage 4**	503	9,226
	8.81%	7.69%
**QC stage 5**	321	6,790
	5.62%	5.66%

Table SM1c. Exclusion regions.

Area	Region 1	Region 2
Immunoglobulin	chr22:20715572-21595082	N/A
Immunoglobulin	chr14:105065301-106352275	N/A
Immunoglobulin	chr2:88937989-89411302	N/A
Immunoglobulin	chr14:21159897-22090937	N/A
Centromere	chr1:121000001-128100000	N/A
Centromere	chr2:90900001-95800000	N/A
Centromere	chr3:89300001-93300000	N/A
Centromere	chr4:48600001-52500000	N/A
Centromere	chr5:45700001-50600000	N/A
Centromere	chr6:58300001-63500000	N/A
Centromere	chr7:57300001-61200000	N/A
Centromere	chr8:43100001-48200000	N/A
Centromere	chr9:46600001-60400000	N/A
Centromere	chr10:38700001-42200000	N/A
Centromere	chr11:51300001-56500000	N/A
Centromere	chr12:33100001-36600000	N/A
Centromere	chr13:13400001-18500000	N/A
Centromere	chr14:13500001-19200000	N/A
Centromere	chr15:14000001-18500000	N/A
Centromere	chr16:34300001-40800000	N/A
Centromere	chr17:22000001-23300000	N/A
Centromere	chr18:15300001-17400000	N/A
Centromere	chr19:26600001-30300000	N/A
Centromere	chr20:25600001-28500000	N/A
Centromere	chr21:9900001-13300000	N/A
Centromere	chr22:9500001-16400000	N/A
Telomere	chr1:1-500000	chr1:246749719-247249719
Telomere	chr2:1-500000	chr2:242451149-242951149
Telomere	chr3:1-500000	chr3:199001827-199501827
Telomere	chr4:1-500000	chr4:190773063-191273063
Telomere	chr5:1-500000	chr5:180357866-180857866
Telomere	chr6:1-500000	chr6:170399992-170899992
Telomere	chr7:1-500000	chr7:158321424-158821424
Telomere	chr8:1-500000	chr8:145774826-146274826
Telomere	chr9:1-500000	chr9:139773252-140273252
Telomere	chr10:1-500000	chr10:134874737-135374737
Telomere	chr11:1-500000	chr11:133952384-134452384
Telomere	chr12:1-500000	chr12:131849534-132349534
Telomere	chr13:1-500000	chr13:113642980-114142980
Telomere	chr14:1-500000	chr14:105868585-106368585
Telomere	chr15:1-500000	chr15:99838915-100338915
Telomere	chr16:1-500000	chr16:88327254-88827254
Telomere	chr17:1-500000	chr17:78274742-78774742
Telomere	chr18:1-500000	chr18:75617153-76117153
Telomere	chr19:1-500000	chr19:63311651-63811651
Telomere	chr20:1-500000	chr20:61935964-62435964
Telomere	chr21:1-500000	chr21:46444323-46944323
Telomere	chr22:1-500000	chr22:49191432-49691432

Table SM2. Platforms and probe coverage at the 16p13.11 locus (Chr16: 14.66-18.70 Mb, GRCh37) for arrays used in cases and controls.

Sample	n	Evaluation platform	Probe coverage	NAHR-med Duplications	NAHR-med Deletions
BB-GRE Cases	10,397	60K Agilent oligo array	49	28	16
Screened Controls	348	Illumina HumanHap 550K	520	0	0
WTCCC2 Controls	4,828	Illumina HumanHap 550K	520	9	3
Shaikh Controls	2,026	Illumina HumanHap 550K	520	3	0
Cooper et al (London controls)	760	Illumina HumanHap 550K	520	0	0
Cooper et al (HGDP controls)	984	Illumina HumanHap 650Y	604	0	0
Cooper et al (FHCRC controls)	1,429	Illumina Quad 610K	625	1	1

Figure SM1. Probe distribution at the 16p13.11 locus (Chr16:14.66-18.70Mb, GRCh37) for arrays used in cases and controls. Despite the lower probe density compared to the Illumina SNP arrays, the Agilent 60K array provides an adequate probe coverage of the three single copy sequence intervals (I, II and III) at 16p13.11-p12.3, appropriate for the detection of the large (>800 kbp) NAHR-mediated 16p13.11 variations, which encompass one or more of the three genomic intervals.

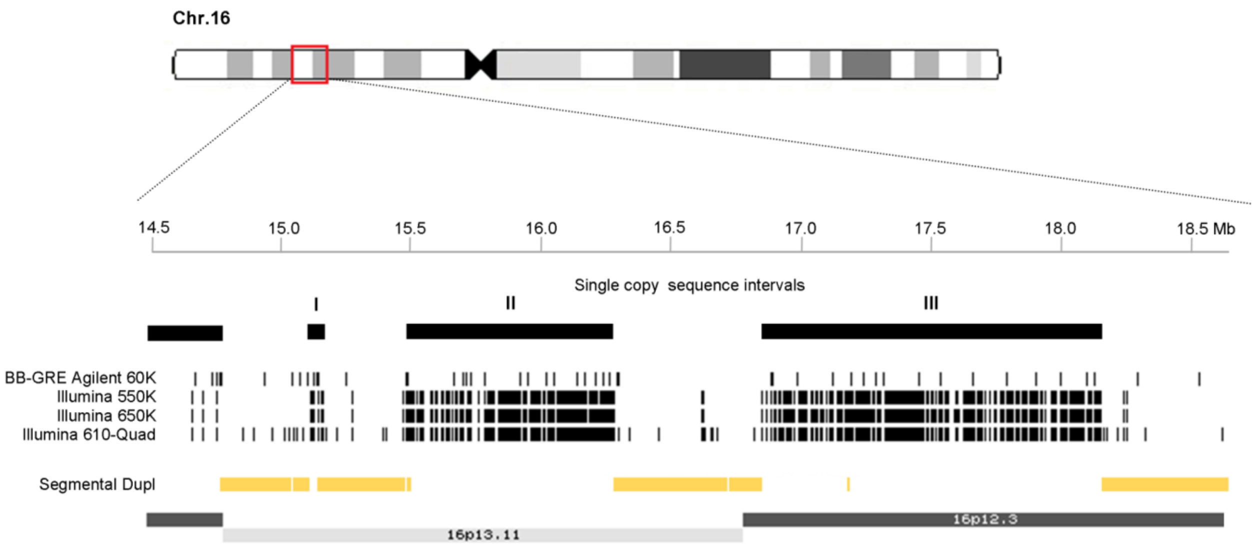

Table SM3. BB-GRE 60K Agilent oligonucleotide array probes at the 16p13.11 locus (Chr16: 14.66-18.70 Mb, GRCh37).

Chr	HG19 start	HG19 stop
chr16	14.680.168	14.680.229
chr16	14.742.264	14.742.325
chr16	14.762.237	14.762.298
chr16	14.771.032	14.771.093
chr16	14.780.133	14.780.194
chr16	14.944.558	14.944.619
chr16	15.048.749	15.048.810
chr16	15.081.352	15.081.412
chr16	15.111.245	15.111.295
chr16	15.131.721	15.131.782
chr16	15.144.118	15.144.179
chr16	15.154.685	15.154.746
chr16	15.256.684	15.256.745
chr16	15.492.315	15.492.372
chr16	15.496.836	15.496.897
chr16	15.677.024	15.677.085
chr16	15.711.354	15.711.415
chr16	15.719.338	15.719.399
chr16	15.740.949	15.741.010
chr16	15.795.562	15.795.623
chr16	15.929.433	15.929.494
chr16	15.960.082	15.960.143
chr16	16.031.653	16.031.714
chr16	16.060.382	16.060.443
chr16	16.148.693	16.148.754
chr16	16.180.944	16.181.005
chr16	16.222.991	16.223.052
chr16	16.249.546	16.249.607
chr16	16.276.054	16.276.115
chr16	16.305.675	16.305.736
chr16	16.311.009	16.311.070
chr16	16.895.833	16.895.894
chr16	16.899.615	16.899.676
chr16	16.999.298	16.999.359
chr16	17.134.562	17.134.623
chr16	17.202.183	17.202.240
chr16	17.250.129	17.250.190
chr16	17.298.052	17.298.113
chr16	17.330.185	17.330.246

chr16	17.468.580	17.468.641
chr16	17.550.935	17.550.996
chr16	17.675.943	17.676.004
chr16	17.804.305	17.804.366
chr16	17.917.640	17.917.701
chr16	18.012.400	18.012.461
chr16	18.112.715	18.112.776
chr16	18.140.990	18.141.051
chr16	18.306.804	18.306.854
chr16	18.546.698	18.546.759
